# Subcutaneous power supply by NIR-II light

**DOI:** 10.1038/s41467-022-34047-5

**Published:** 2022-11-03

**Authors:** Shanzhi Lyu, Yonglin He, Xinglei Tao, Yuge Yao, Xiangyi Huang, Yingchao Ma, Zhimin Peng, Yanjun Ding, Yapei Wang

**Affiliations:** 1 Key Laboratory of Advanced Light Conversion Materials and Biophotonics, Department of Chemistry, Renmin University of China, 100872 Beijing, China; 2 Department of Energy and Power Engineering, Tsinghua University, 100082 Beijing, China

**Keywords:** Devices for energy harvesting, Biomedical engineering

## Abstract

Implantable medical devices are wished to be recharged via contactless power transfer technologies without interventional operations. Superior to subcutaneous power supply by visible light or electromagnetic wave, second near-infrared (NIR-II) light is predicted to possess 60 times subcutaneous power transmission but hard to be utilized. Here we report a photo-thermal-electric converter via the combination of photothermal conversion and thermoelectric conversion. It is able to generate an output power as high as 195 mW under the coverage of excised tissues, presenting advantages of non-invasion, high output power, negligible biological damage, and deep tissue penetration. As an in vivo demonstration, the output power of a packaged converter in the abdominal cavity of a rabbit reaches 20 mW under NIR-II light irradiation through the rabbit skin with a thickness of 8.5 mm. This value is high enough to recharge an implanted high-power-consumption wireless camera and transfer video signal out of body in real-time.

## Abstract (web summary)

The authors present a subcutaneous power supply which coverts NIR-II light to electricity via combination of photothermal conversion and thermoelectric conversion. They demonstrate that it can recharge an implanted wireless camera upon irradiation with NIR-II light through the skin.

## Introduction

**Fig. 1:**
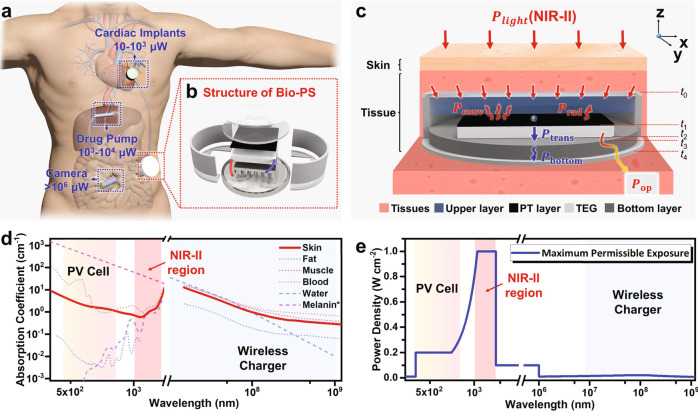
The subcutaneous power supply by NIR-II light. **a** The power consumption of clinically used IMDs and the imagination of recharging those batteries in the IMDs by a CPT device. **b** The disassembled structure of a subcutaneous power supply device made of a tandem combination of the photothermal converter and thermoelectric converter. **c** The heat transfer model of PTE converter under NIR-II light irradiation. **d** The absorption coefficient of light with different wavelengths and electromagnetic radiation (10^2^−10^9^ nm) by the biological constituents within skin tissues. The absorption curve of melanin is obtained by fitting calculations of some known values. **e** The maximum permissible exposure power density of the skin tissues to light with different wavelengths and electromagnetic radiation in unrestricted environments (10^2^−10^9^ nm).

Implantable medical devices (IMDs) with electrical intervention appear to be an innovative branch of restorative treatments and clinical diagnosis. For instance, the death rate caused by heart failure has been lowered by 80% by means of cardiac implants^1,2^; Permanent implant of electrical stimulators affords great promise to decrease the mortality and even regain movement of patients with spinal cord injuries^3^; The invention of the implanted medical sensor, such as laparoscope, could determine the position, range and transfer of chronic pancreatitis, diabetes, or ovarian cancer in real time^4–6^. However, clinical IMDs to date are mainly powered by batteries, which are faced with shortages of limited energy capacity^7^. Surgical replacement of battery has to be frequently needed in the case of an exhausted power supply. The limitation of power supply also impedes the development of IMDs with more complicated functions that need higher power consumption, e.g. from several microwatts to hundreds of milliwatts^8,9^ (Fig. 1a). Therefore, prolonging the lifetime of power supply is rising as one subject of intense investigation and consideration in developing IMDs^10–12^.

Contactless power transfer (CPT) would be an ideal complement to the IMDs with battery-only power supply, which is anticipated to avoid the risk of surgical replacement. In principle, CPT enables the exhausted batteries to be recharged in situ by virtue of energy conversion through skin tissues. In order to satisfy a wide application of IMDs, the charging power led by CPT technology needs to exceed 10 mW with a transmission depth through skin tissues of more than 10 mm, and meanwhile, the clinically relevant CPT should not cause injuries to tissues or organs during the power transfer process. From our survey, two types of CPT technologies have been exploited for subcutaneous power supply, including electromagnetic coupling using electromagnetic waves (300 MHz–20 GHz)^13,14^ and photovoltaics using visible light^15^. However, there is no successful CPT technology that has been clinically applied so far owing to the limitation of outpower or transmission depth.

Superior to visible light, the second near-infrared (NIR-II) light with a wavelength ranging from 1000 to 1350 nm, has been recognized as a biologically transparent window, in light of less absorption and scattering by skin tissues^16–20^ (Fig. 1b and Supplementary Fig. S1). Concretely, 99% of visible light energy would be absorbed by the tissues underneath the skin <2 mm. NIR-II light exhibits much deeper penetration through skin tissues (at least 20 mm) and also causes minimal damage to tissues than light with other wavelengths^21,22^. As specifically estimated in Fig. 1e, human skins can endure NIR-II light irradiation at a maximum power density of 1.0 W cm^−2^, without the appearance of evident tissue damage. However, in unrestricted environments, the maximum permissible exposure power density under irradiation by visible light and electromagnetic wave (300 MHz–20 GHz) are only allowed below 0.2 and 0.02 mW cm^−2^, respectively^23,24^. For the same subcutaneous tissue, the theoretical maximum endurance energy of NIR-II is 60 times higher than visible light and electromagnetic waves, respectively. In regard to these aspects, NIR-II light is attracting significant interest in biomedical applications, particularly focused on therapeutic treatment and biological imaging. However, those successful examples were mainly attributed to the principles of photothermal conversion or emissive fluorescence. In spite of deep tissue penetration, NIR-II light was rarely considered in the design of photovoltaic cells for clinical CPTs, as photoelectric conversion efficiency is significantly limited by the poor exciton separation^25–28^.

Here, we invented a CPT technology with appreciable output power by NIR-II light. As presented in Fig. 1b, after passing through the skin tissue, NIR-II light is absorbed by the photothermal layer (PT layer), accordingly generating photothermal heat which is quickly transferred to a thermoelectric generator (TE generator) underneath the PT layer, the whole process could be called as photo-thermal-electric conversion (PTE). The three-layer structure is established and further optimized according to the theoretical suggestions by a heat transfer model (Fig. 1c). Following this concept and the safety standard, in a simulation test of covering the PTE converter with a piece of porcine skin tissue with a thickness of 10 mm, an output power higher than 10 mW is successfully achieved. Referring to published literature and the safety standard, such a power is the highest value among all CPTs applied in subcutaneous power supply. It is high enough to drive a heart pacemaker and even a camera, which offers great promise for recharging those powerless IMDs without the need for repeated surgical operations.

## Result

### The heat transfer model involved in a PTE converter

Referring to the model as presented in Fig. 1c, the PTE converter that is implanted underneath a skin tissue consists of an upper layer, a TE generator coating with a photothermal layer (PT layer), a bottom layer, and a biocompatible shell to enclose all energy transfer parts. During the irradiation process, the output power is supposed to reach maximum whenever the system approaches heat transfer balance. If the external encapsulated shell is tangent to the TE generator, the heat loss in the horizontal direction could be significantly inhibited. Thus the heat mainly transfers in the vertical direction along incident light under the condition of confined space, which obeys a one-dimension steady-state heat transfer model^29,30^ (see the Supplementary Discussion). Those symbols in the model are specifically explained in Supplementary Table 1. According to the principle of energy conservation, an equation related to the PT layer is established in Eq. (1),

1 $${P}_{{{\rm {PT}}}}-{P}_{{{\rm {loss}}}}={P}_{{{\rm {trans}}}}+{P}_{{{\rm {op}}}}$$

where *P*_PT_ is the power of photothermal conversion and is regarded as the input energy, *P*_loss_ represents the heat loss of the PT layer and it accounts for the hyperthermia effect on tissues above the PTE converter. *P*_trans_ means the power transferred from the PT layer to the TE generator for thermoelectric conversion, which is defined by (*t*_1_−*t*_2_)*λ/*D*, in which *t*_1_ and *t*_2_ are the top surface temperature and bottom surface temperature of the TE generator; *λ* and *D* are the thermal conductivity and thickness of TE generator. *P*_op_ refers to the maximum output power of thermoelectric conversion by TE generator which is described by Eq. (2):

2 $${P}_{{{\rm {op}}}}=\frac{{S}^{2}\left(t\right){({t}_{1}-{t}_{2})}^{2}}{R(t)}$$

where *S*(*t*) is the Seebeck coefficient and *R*(*t*) is the inherent resistance of the TE generator, which are related to the average temperature and properties of TE materials. According to Eq. (2), besides decreasing *R*(*t*) and increasing *S*(*t*), rational heat management is crucially important to enlarge the temperature difference between the PT layer and the bottom surface of the TE generator so as to obtain higher *P*_op_.

The upward thermal transfer of *P*_loss_ which is an unfavorable factor for photoelectric conversion is induced by thermal conduction, thermal convection, and thermal radiation.

3 $${P}_{{{\rm {loss}}}}={P}_{{{\rm {conv}}}}+{P}_{{{\rm {rad}}}}={A}_{1}*{h}_{1}*\left({t}_{1}-{t}_{0}\right)+\frac{\sigma \left[{t}_{1}^{4}-{t}_{0}^{4}\right]}{\frac{1}{{\varepsilon }_{1}}+\frac{(1-{\varepsilon }_{2}){A}_{0}}{{\varepsilon }_{2}{A}_{1}}}$$

*P*_conv_ refers to a comprehensive heat loss of the PT layer resulting from thermal conduction and convection, and *P*_rad_ is the heat loss by thermal radiation. In Eq. (3), *A*_1_ is the area of the PT layer, *h*_1_ represents the convective heat transfer coefficient, *σ* is the Stefan–Boltzmann constant, *t*_0_ is the temperature of the tissue in contact with the top surface of the PTE converter, and *ε*_1_, *ε*_2_ are the emissivity of PT layer and tissue, respectively. To ensure the safety of the PTE converter, *t*_0_ should be around 37 °C during light irradiation. *P*_rad_ can be Taylor expanded and approximated as a linear function of *t*_1_−*t*_0_, and thus Eq. (3) is simplified as

4 $${P}_{{{\rm {loss}}}}={A}_{1}*{H}_{1}*({t}_{1}-{t}_{0})$$

*H*_1_ is equivalent to the heat loss coefficient combining heat convection and radiation.

5 $${P}_{{{\rm {bottom}}}}={P}_{{{\rm {trans}}}}={A}_{2}*{H}_{2}*({t}_{2}-{t}_{3})$$

where *P*_bottom_ is the downward thermal transfer from the bottom surface of the TE generator, *A*_2_ and *H*_2_ are the heat transfer area and coefficient of the filling medium between the bottom surface of the TE generator and the top surface of the bottom layer, respectively. *t*_3_ is the temperature at the top surface of the bottom layer which is higher than *t*_0_. So *t*_1_−*t*_2_ can be figured out by Eq. (6),

6 $${t}_{1}-{t}_{2}=\frac{{P}_{{{{{{\rm{PT}}}}}}}-{H}_{1}{A}_{1}({t}_{3}-{t}_{0})}{\left(\frac{{H}_{1}{A}_{1}}{{H}_{2}{A}_{2}}+1\right)\frac{\lambda }{D}+{H}_{1}{A}_{1}}$$

Therefore, enlarging *t*_1_−*t*_2_ can be achieved by reducing the heat loss (*H*_1_A_1_), intensifying the heat transfer between the bottom surface and the lower encapsulating layer (*H*_2_A_2_), increasing *P*_PT_, and keeping *t*_3_ at a low temperature. It should be noted that analysis by finite-element model or non-steady-state heat transfer model gave the same factors that closely affect *t*_1_−*t*_2_ (Supplementary Figs. S2–5, Movies S1–3 and Supplementary Discussion).

### The preparation and characterization of PT layer

The thermal sponsor of *P*_PT_ is determined by the power of incident light (*P*_light_), photothermal conversion efficiency (*η*_PT_), and light transmittance of skin tissues (*T*).

7 $${P}_{{PT}}=T{\eta }_{{{{{{\rm{PT}}}}}}}{P}_{{{{{{\rm{light}}}}}}}$$

**Fig. 2:**
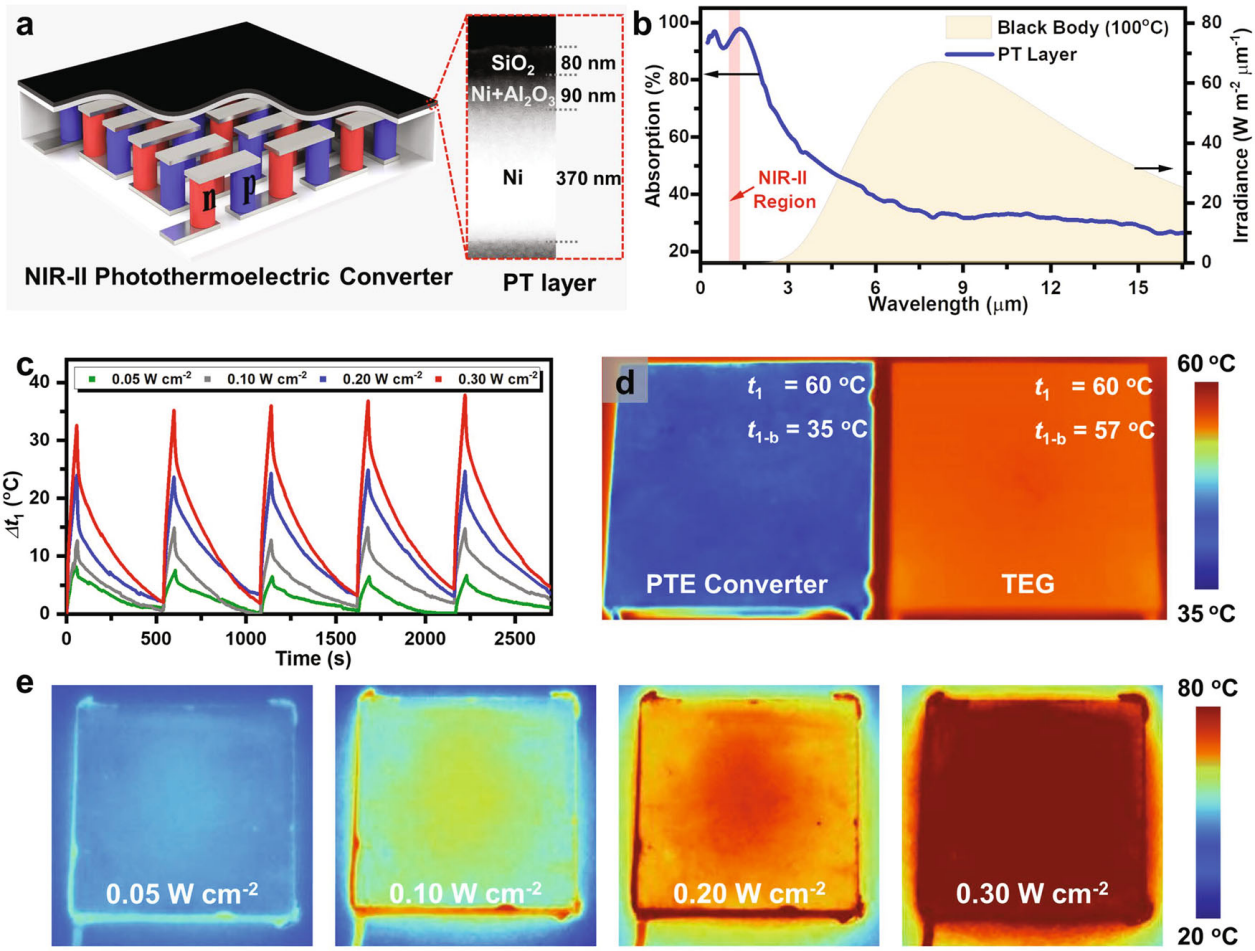
The design and characterization of PT layer. **a** Schematic of the composition of the PT layer. Inset: corresponding scanning electron microscope image of the PT layer, which consists of three layers, from top to bottom including an 80 nm of SiO_2_, a 90-nm-thick Ni/Al_2_O_3_ complex layer, and a 370-mm-thick Ni substrate. **b** The absorption spectrum of PT layer, and its comparison with the irradiance from a blackbody at 100 °C. **c** The temperature changes of the PT layer coating on a TE generator under cycles of on–off NIR-II light irradiation with different LPDs. The different LPT of 0.05, 0.10, 0.20, 0.30 W cm^−2^ corresponding to skin tissues with different transmittance of 5%, 10%, 20%, 30%, respectively. **d** The infrared image (IR image) representing the brightness temperature of PT layer coating on TEG which is placed on a hot platform whose temperature is 60 °C. **e** The IR image of PT layer coating on TE generator under NIR-II light irradiation with different LPDs for 10 min.

Reducing the loss of light energy passing through the skin tissue can generate higher *P*_PT_ at a given *P*_light_, which explains the choice of NIR-II light. *η*_PT_ is an inherent attribute of NIR-II light absorber and it is a critical determinant of *P*_PT_. The PT layer was composed of a SiO_2_ and a Ni/Al_2_O_3_ complex layer on a Ni substrate^31^ (Fig. 2a, Supplementary Fig. S6), in which the SiO_2_ layer is beneficial for antireflection and the Ni/Al_2_O_3_ layer accounts for the intense absorption of NIR-II light as well as low thermal emittance at high temperature (Fig. 2b). Owing to the reinforcement of light absorption by Ni substrate, the PT layer has an extraordinary *η*_PT_ about 94%^32^ (Supplementary Fig. S7 and see the section “Discussion”). It is worth noting that the radiation heat loss of PT layer on a hot plate was indistinctive due to its low thermal emittance *ε*_1_, which is in agreement with the low-temperature distribution observed by the infrared camera (Fig. 2d). The PT layer was placed under NIR-II light irradiation with different light power densities (LPDs) for 10 min to simulate the varied NIR-II light transmittance through skin tissues with different thickness. Accordingly, Δ*t*_1_ were raised to 13.7 °C (*T* = 5%), 27.5 °C (*T* = 10%), 49.3 °C (*T* = 20%), and 70.5 °C (*T* = 30%) (Fig. 2e, Supplementary Fig. S8–9). Meanwhile, the PT layer exhibits adequate stability and durability against cycled light irradiation according to the fully repeated temperature change under on–off NIR-II light irradiation (Fig. 2c, Supplementary Fig. S10).

### The design and characterization of the upper layer

**Fig. 3:**
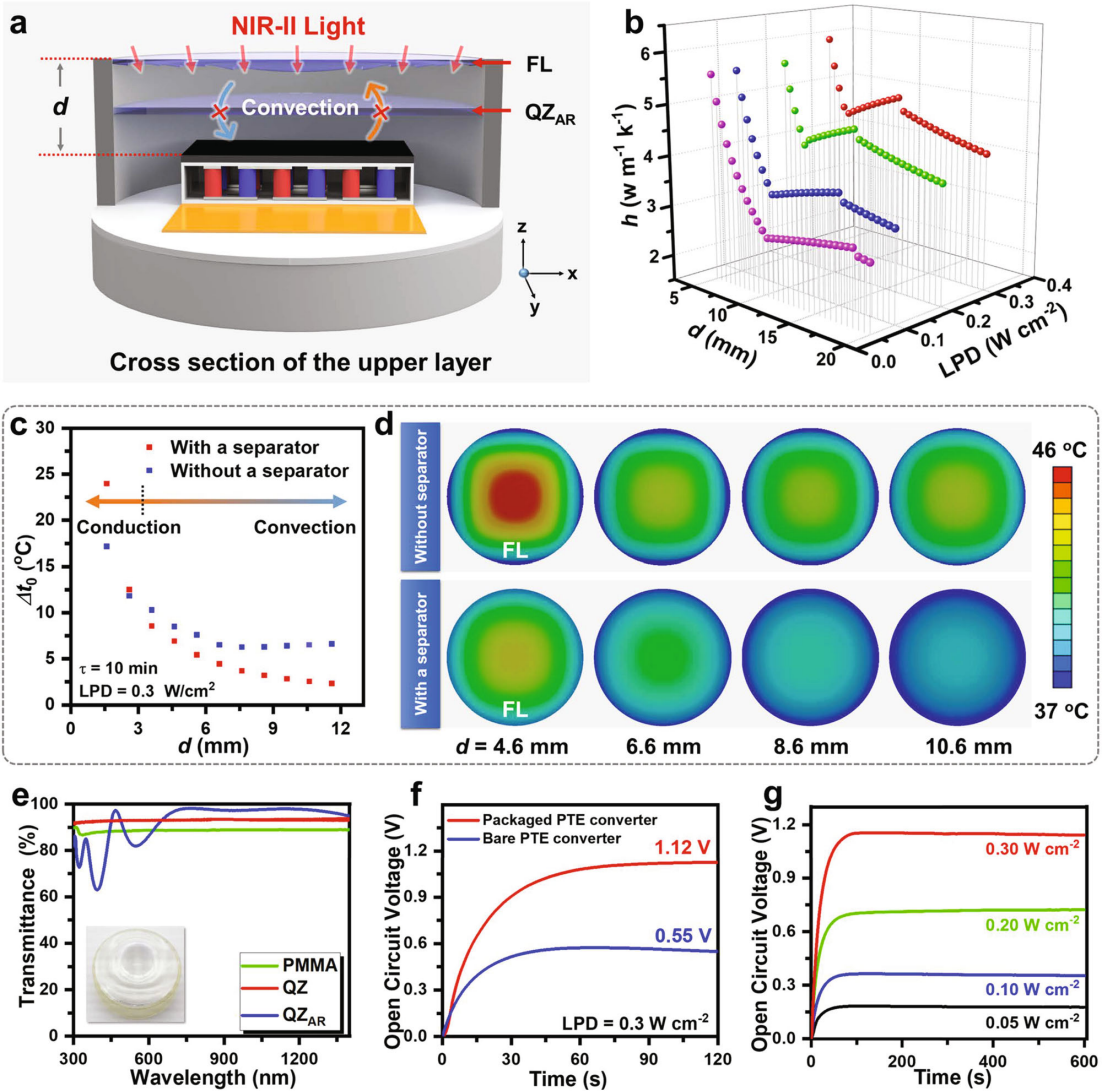
The design and characterization of the upper layer. **a** Schematic of the cross-section of the upper layer, from top to bottom, including a Fresnel lens, an air layer separated by a transparent quartz plate with antireflection coatings, PT layer on the top of a TE generator. The entire PTE converter is enclosed by a polymethyl methacrylate shell. **b** The estimation of convective heat transfer coefficient in the presence of an air layer with different *d* between PT layer and Fresnel lens, or under NIR-II light irradiation with different LPDs. **c** The investigation of the effect of the separator inserted in the air layer with different *d*, by recording Δ*t*_0_ relative to body temperature (37 °C) under NIR-II irradiation with LPD of 0.3 W cm^−2^ for 10 min. **d** The predicted temperature distribution on the Fresnel lens which is the top surface of the PTE converter under NIR-II irradiation with LPD of 0.3 W cm^−2^ for 10 min via finite element analysis. The top images are assigned to the upper layer without or with a quartz separator, respectively. **e** The transmittance of PMMA (raw materials of Fresnel lens and protective shell), the quartz separator, and the quartz separator with antireflective coatings in VIS–NIR window. The thickness of each sample is 1.0 mm. Inset: the image of Bio-PS. **f** The time-dependent open circuit voltage of the bare PTE converter and packaged PTE converter having an upper layer under NIR-II light irradiation with LPD of 0.3 W cm^−2^. **g** Open circuit voltage generated by PTE converter with an upper layer under NIR-II light irradiation with different LPDs.

As illustrated in Fig. 3a, on the top of the PT layer are there an air layer and a Fresnel lens in turn. The air layer could balance *T* and low thermal conductivity, and the latter serves as a top cap of the whole PTE converter and is also used to amplify *P*_light_. Increasing the thickness of the air layer (*d*) is not always positive to lowering heat loss due to the consequent deterioration of heat convection (Supplementary Fig. S11). As summarized in Fig. 3b, *h*_1_ in Eq. (3) is closely related to the thickness of the air layer and light power density. Distinctly, *h*_1_ obeys a trend of decrease and then rise before and after a critical thickness of air layer (*d*_c_). The first stage satisfies the effect of thermal insulation by the air layer and the air convection plays a leading role in heat transfer when the air thickness exceeds *d*_c._ On the other hand, increasing the temperature of the PT layer, corresponding to higher *t*_1_, will lower *d*_c_ value because of the acceleration of thermal convection. This principle explains the appearance of decreased *d*_c_ under NIR-II light irradiation with higher LPD. Practically, to suppress the heat convection led by a thicker air layer, the air layer is separated by a transparent quartz plate deposited with anti-reflective coatings^33^. This separator allows more than 99% of NIR-II light to pass through it while significantly reducing *P*_conv_ and *P*_rad_ between the PT layer and the Fresnel lens and further reducing *P*_loss_. As predicted by finite-element analysis, the insertion of a separator in the air layer distinctly affects the temperature change on the Fresnel lens (Δ*t*_0_) relative to body temperature (37 °C) (Fig. 3c, d, and Supplementary Figs. S12–14). Δ*t*_0_ is lowered in the presence of a quartz plate separator when *d* is >3 mm, indicating that heat transfer from the PT layer to the Fresnel lens is successfully minimized. Giving comprehensive consideration to heat transfer and the focus of the Fresnel lens, *d* is fixed as 8.6 mm in the following investigations (Fig. 3e). In this case, Δ*t*_0_ is <5 °C under NIR-II light irradiation with LPD of 0.3 W cm^−2^ for 10 min. Noting the demands on biocompatibility, the external shell, and Fresnel lens that contact with surrounding tissues were both made of polymethyl methacrylate. It is ensured that less than 10% of NIR-II light is consumed by the Fresnel lens and the upper layer before it reaches the PT layer, meanwhile, more than 70% of heat loss is reduced by them (Fig. 3e and Supplementary Figs. S15–16).

The PTE converter having an upper layer outputs a maximum open circuit voltage of 1.12 V under NIR-II irradiation with LPD of 0.3 W cm^−2^, twice greater than PTE without an upper layer (Fig. 3f). It was observed that the open circuit voltage resulting from the PTE converter without upper layer was declined over time after reaching a maximum (Supplementary Figs. S17–18), which should be caused by the enhanced *P*_loss_ at a higher temperature, which was also consistent with the results of non-steady-state heat transfer model. In comparison, since the heat transfer is suppressed by the upper layer, the open circuit voltages generated by the PTE converter having an upper layer could be held unchanged after reaching the maximum state, positively depending on the LPDs (Fig. 3g).

### The design and characterization of bottom layer

**Fig. 4:**
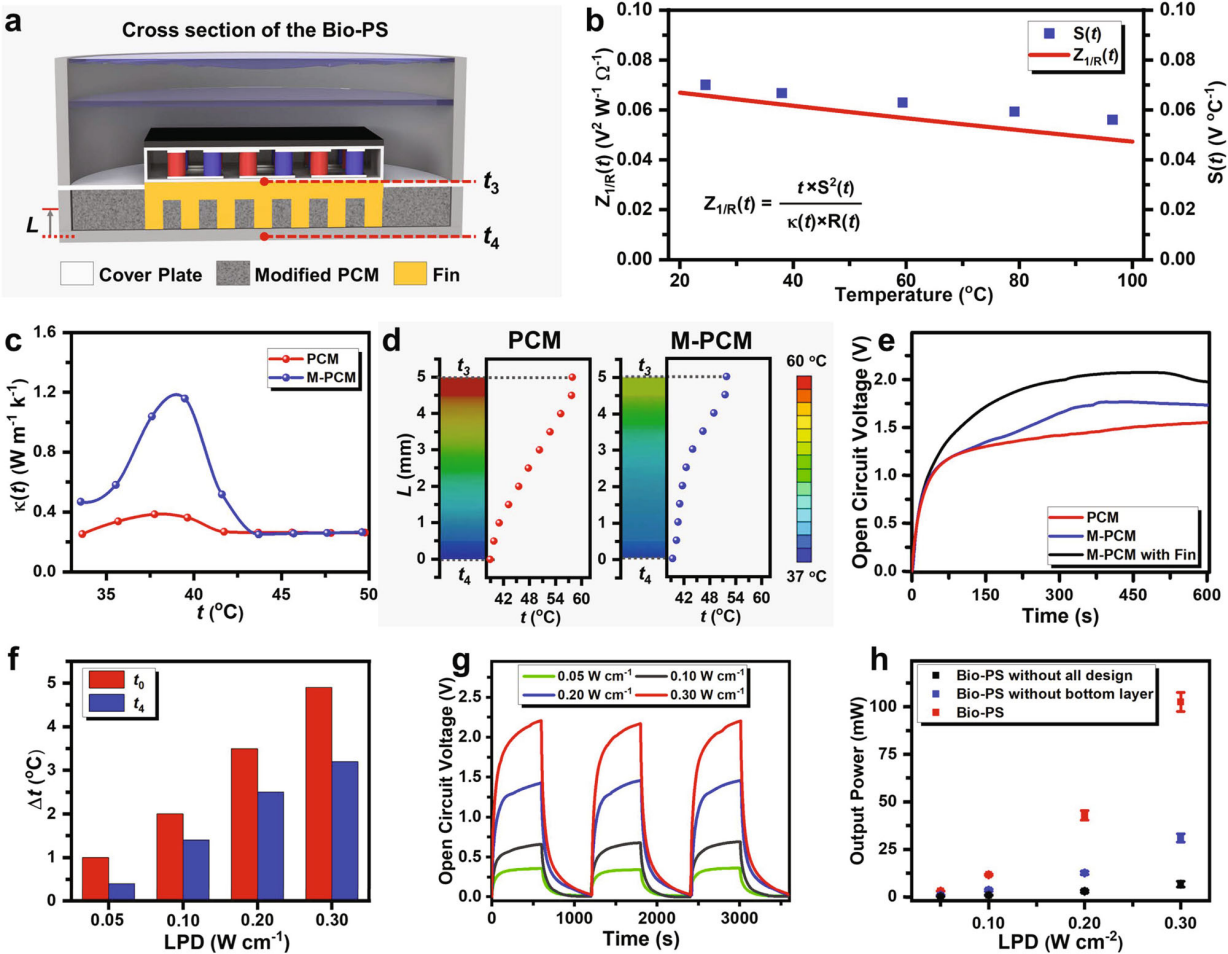
The design of the bottom layer, and the characterization as well as safety test of Bio-PS. **a** Schematic illustration of the cross-section of the bottom layer. **b** Thermoelectric figure of merit (*Z*_1/R_(*t*), thermoelectric figure of merit defined by resistance instead of electrical conductivity and Seebeck coefficient (*S*(*t*)) of TE generator at different qualitative temperatures. **c** Thermal conductivity of the phase change material (PCM) and black carbon-modified PCM (M-PCM) at varied temperatures. **d** The temperature distribution through the center of the bottom layer under constant heat flow for 10 min predicted by Ansys, and the statistical curve of temperature with different lengths of PCMs (*L)* The heat flux was 2.0 W. **e** Time-dependent open circuit voltage output by TE generator with the bottom layer of PCM (red line), the modified PCM (blue line), and the combination of modified PCM with cooling fin (black line). The slight decrease in the open circuit voltage resulted from the fluctuation of temperature during the phase transition of PCMs. **f** The determination of temperature change relative to body temperature (37 °C) on the upper and lower surfaces of the packaged Bio-PS, under NIR-II light irradiation with LPD of 0.30 W cm^−2^ for 10 min. **g** The changes of open circuit voltage of Bio-PS over the cycles of on–off NIR-II light irradiation with the different LPDs. **h**, The comparison of maximum output power between the PTE converters with or without the use of the bottom layer under NIR-II light irradiation with different LPDs.

Referring to the heat transfer model as stated above, the bottom layer underneath the TE generator is expected to have a large special heat capacity and heat transfer coefficient so as to increase *H*_2_*A*_2_ as well as keep *t*_3_ at a low temperature. As illustrated in Fig. 4a, a cooling fin, which could increase the area of surface-heat transfer *A*_2_, together with phase change material (PCM) was bound to the bottom of the TE generator in order to lower *t*_3_. Moreover, both the *S*(*t*) and *Z*_1/*R*_(*t*) decrease as the qualitative temperature (defined as (*t*_1_ + *t*_2_)/2) rises (Fig. 4b, Supplementary Fig. S20). This observation is in agreement with the increased *R* at higher temperatures by increasing the NIR-II light power density in Supplementary Fig. S19. It could further reduce *P*_op_ theoretically (Eq. (2)). In practice, the cooling fin was filled and enclosed by myristyl alcohol which was used as the PCM in terms of its phase transition temperature close to body temperature, in order to dissipate the thermal energy at the bottom of TE generator and thus decrease *t*_2_ (Supplementary Discussion). It is anticipated that Seebeck voltage as well as thermoelectric figure of merit would be improved at lower *t*_2_. To avoid the leakage of the PCMs when melted, an aluminum alloy base was built in the external resin shell so as to prevent PCM from contacting the resin shell directly.

Myristyl alcohol with the addition of carbon black was used as PCM, whose thermal conductivity is higher than pure one but its specific heat capacity is less^34^ (Fig. 4c and Supplementary Figs. S21–22). Regarding this conflict, the comprehensive effect of carbon black-modified PCM was evaluated by finite element analysis (Fig. 4d and Supplementary Figs. S23–25). The simulation predicts that the modified PCM can decrease *t*_3_ by 6 °C more than that of pure PCM, though the temperature on the bottom surface of the external shell (*t*_4_) by modified PCM is only 0.5 °C higher than *t*_4_ in the case of pure PCM. According to the distinctly improved thermoelectric voltage, the cooling fin is necessarily needed to enlarge the thermal exchanging area between the TE generator and modified PCM (Figs. 4e and S26, S27).

### The characterization and safety evaluation of Bio-PS

We name the total packaged energy transfer device as Bio-Power Supply (Bio-PS). As recorded in Fig. 4f and Supplementary Figs. S28–29, the increase of *t*_0_ and *t*_4_ relative to body temperature was both <5 °C over the course of 10-min NIR-II light irradiation with different LPDs. It is ensured that the Bio-PS would not cause hyperthermia to the tissues closely contacted with it. Besides providing safety promise to tissues underneath the Bio-PS, the sufficient heat storage by the bottom layer also raised the open circuit voltages of the PTE converter (Fig. 4g), in contrast to the PTE converter only with the upper layer (Fig. 3g). For example, under NIR-II light irradiation with LPD of 0.3 W cm^−2^, the open circuit voltage reached to 2.21 V with the use of upper layer and the bottom layer at the same time, yet an open circuit voltage of 1.12 V was obtained for the case using upper layer only. When the Bio-PS is connected to a circuit load, it outputs considerable power under NIR-II light irradiation. According to the in vitro studies, the Bio-PS covered by 20 mm-thickness pork, 8.5 mm-thickness the rabbit’s abdomen, or 3.5 mm-thickness porcine skin tissue can output power of 6, 28, or 195 mW, respectively (Supplementary Figs. S30–31). In the project reported here, the multi-layered structure is so important that the energy output efficiency has been raised nearly 20-fold from a PTE converter without other assistant layers to the packaged Bio-PS.

**Fig. 5:**
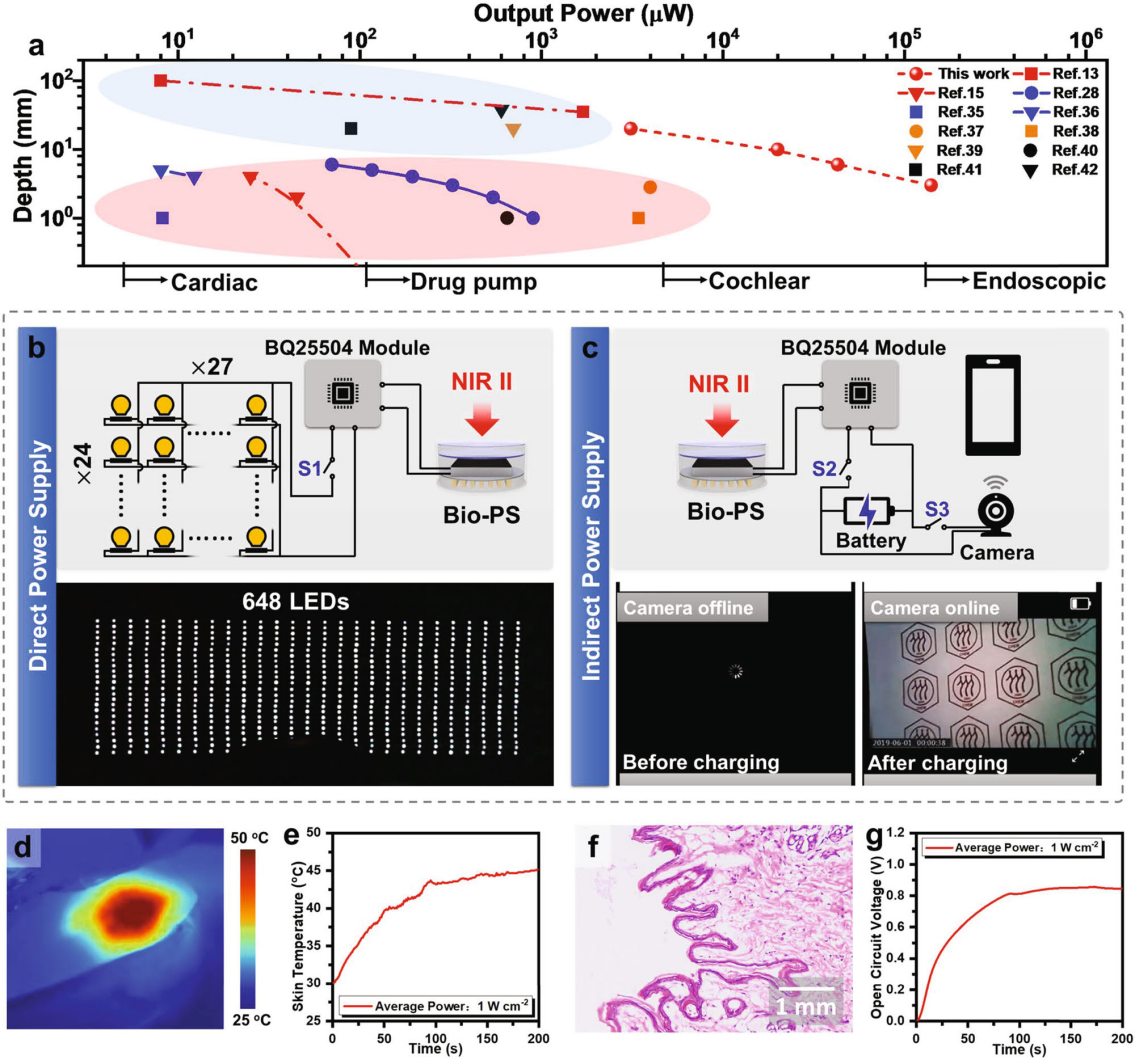
In vitro and in vivo assessment of Bio-PS. **a** The subcutaneous power supply performance of Bio-PS and other CPTs. The data in the red and blue circles correspond to the PV and wireless power transfer, respectively. **b** A demonstration of direct power supply for lighting up light-emitting diodes (LEDs) by Bio-PS. Top: The diagram of the electrical connection between Bio-PS and LEDs (array of 24*27). Bottom: The photograph of 648 LEDs lightened by a Bio-PS under the coverage of 3.5 mm-pigskin under NIR-II light irradiation for 10 s under (LPD of 1.0 W cm^−2^). **c** A demonstration of indirect power supply for recharging and further driving a camera by Bio-PS. Top: The diagram of the electrical connection between Bio-PS, battery, and camera with Wifi. Bottom: The photographs of the camera being offline before charging and the camera being online after charging. **d** The temperature distribution as timely recorded by IR camera on a rabbit skin under NIR-II light irradiation for 200 s (LDP of 1.0 W cm^−2^). **e** The time-dependent skin temperature over the course of NIR-II light irradiation. **f** Standard H&E staining of tissues of rabbit skin that specifically receive NIR-II light irradiation (*n* = 3). **g** The curves of open circuit voltage over time during irradiation.

As compared in Fig. 5a, Bio-PS ranks as the most promising subcutaneous power supply method among the existing CPTs, in consideration of its higher output power at 10 mm underneath skin tissue^35–42^. Such a high output power even over 200 mW offers fascinating promise to drive those IMDs with higher energy consumption, e.g. cochlear, and endoscopic. To demonstrate its promising subcutaneous power supply performance, a Bio-PS covered by a piece of porcine skin tissue with a thickness of 3.5 mm could light up 648 white LEDs under NIR-II light irradiation (Fig. 4b and Supplementary Fig. S32, Movie S4). Using the special step-up transformer (based on BQ25504, its circuit diagram in Supplementary Fig. S33) of stepping up the output voltage to 4.2 V, the same setup could also recharge a power-exhausted camera via the way of subcutaneous power supply (Fig. 4c, Movie S5), and extended to other applications such as charging button battery or super-capacitor, driving the electric fan, lighting up the high power LEDs in different colors with RUC pattern (Supplementary Figs. S34–37, and Movie S6).

### The tests and applications of Bio-PS in vivo

**Fig. 6:**
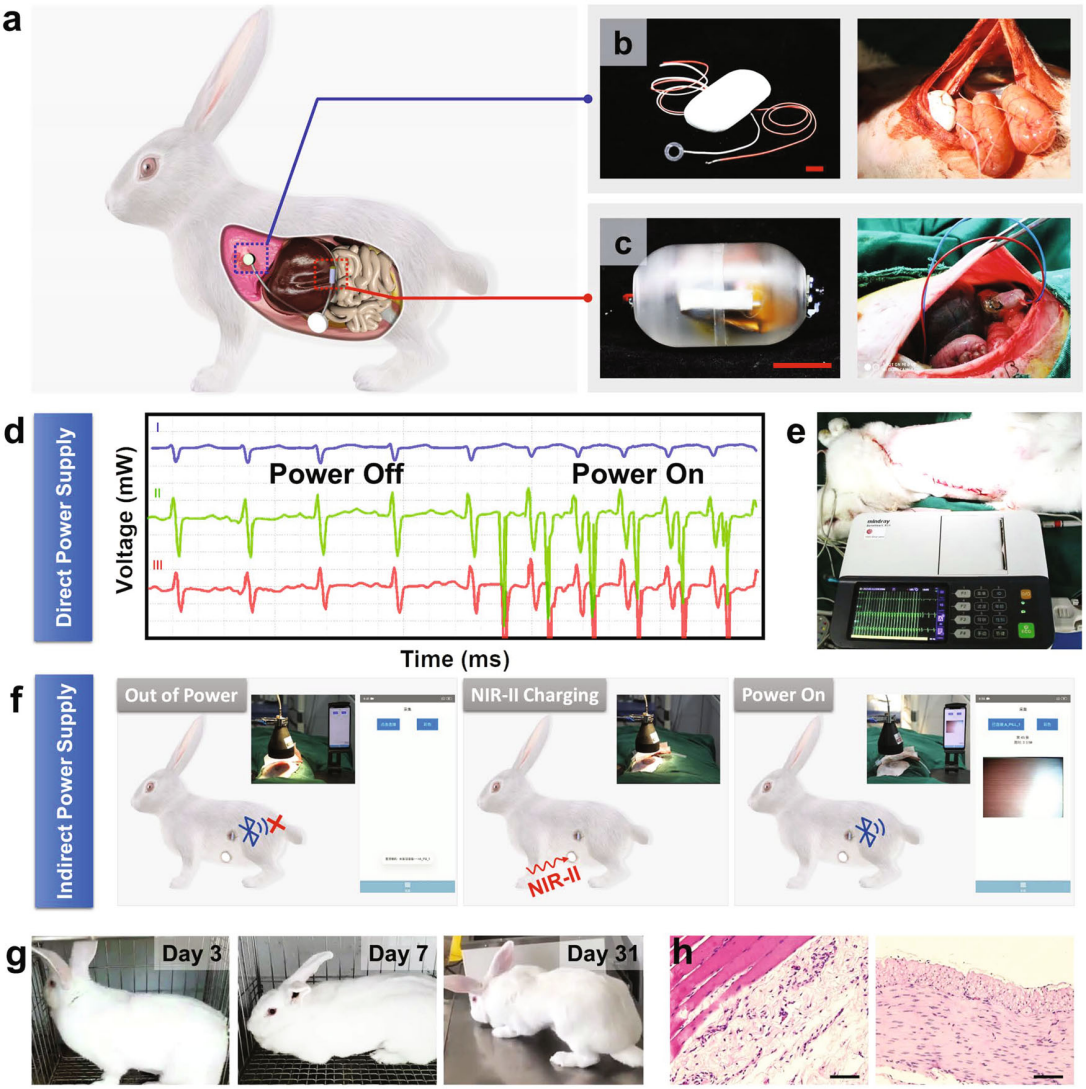
The power supply provided by Bio-PS for IMDs in rabbits. **a** Schematic of the corporation of Bio-PS and IMDs that are both implanted in rabbits. **b** Photograph of a cardiac pacemaker, and its placement in rabbit (inset). Scale Bar: 1.0 cm. **c** Photograph of endo-photo camera that transmits images by Bluetooth, and its placement in rabbit (inset). Scale Bar: 1.0 cm. **d** Electrocardiogram recorded under on–off irradiation. **e** Photograph of cardiac pacemaker powered by Bio-PS under NIR-II light irradiation. **f** The process of indirect power supply via Bio-PS for the endo-photo camera, and corresponding immediate imaging (inset). The screenshots represent the failed connection before charging (left), and the output video transmitted by Bluetooth (right). The video lasts more than 4 min. **g** The living state of the rabbit after the implantation operation. From left to right: Day 3 (Normal eating and exercise had been resumed), Day 7, Day 18, and Day 31, respectively. **h** Standard H&E staining of abdomen tissues after 31 days of implanted surgery. Above (left image) and below (right image) the site of device implantation (*n* = 3). Scale bar: 100 μm.

Bio-PS was placed in the abdominal cavity of rabbits to verify the feasibility of subcutaneous power supply by NIR-II Light in vivo (Supplementary Fig. S38). In the case of subcutaneous tissue with a thickness of 8.5 mm, the skin temperature rose to 45 °C under NIR light irradiation with LPD of 1.0 W cm^−2^ for 200 s (Fig. 5d, e). According to the standard H&E-staining results, the increased temperature led by NIR light irradiation is safe for skin tissues as the irradiated zone and non-irradiated zone are almost the same without evidence of basophilic changes or cuticle damage^43^ (Fig. 5f, Supplementary Fig. S39). Bio-PS implanted within the rabbit body outputs an open circuit voltage of 0.85 V, corresponding to an output power of 20 mW (Fig. 5g). In principle, such a high output power is readily applicable to provide power for IMDs. As illustrated in Fig. 5a–c, the Bio-PS connected with a step-up transformer could successfully charge a clinical cardiac pacemaker or endo-photo camera in vivo. Their specific circuit diagrams were supplied in Supplementary Fig. S41. The electrocardiogram as recorded in Fig. 6d was interfered with by a battery-free pacemaker that was connected to Bio-PS. Placed at the epicardium of the rabbit, the pacemaker could notably cause the change of electrocardiogram, especially in ST-segment once Bio-PS receives NIR-II irradiation (Fig. 6e, Movie S7). It is thus confirmed that Bio-PS is able to afford sufficient energy to power clinical pacemakers.

The endo-photo camera as presented in Fig. 6c needs a higher power consumption (*P*_max_ = 100 mW, Supplementary Fig. S42). These kinds of cameras have done doctors great favors to determine the location of lesions and the extent of deterioration. So far, clinical endoscopic cameras rely heavily on electrical cables or capsule gastroscopes that can only work in a short diagnostic period. Bio-PS is recommended as a sustainable power supplier with minimizing the need for electrical cables. As shown in Fig. 6f, Supplementary Fig. S43, and Movie S8, a millimeter-sized camera having a battery was connected to Bio-PS and both of them were implanted in the rabbit’s abdominal cavity. The battery could be charged by Bio-PS under NIR-II light irradiation and then discharge electricity to the camera controlled by a magnetic switch. Images captured by the camera were received by a mobile phone via Bluetooth. The video lasting 5 min which records the abdominal conditions in real time was obtained after the battery was charged by Bio-PS for 2 h. It is noteworthy that the implantation of Bio-PS did not lead to dramatic negative effects on rabbit’s life. The rabbit was able to eat food and drink water as normal on the second day after implantation surgery. It did not exhibit abnormal symptoms since the implanting operation for 4 weeks (Fig. 6g, Movie S9). Further histological H&E-staining analysis indicated that ignorable destroys were found within subcutaneous tissues behind Bio-PS and intestinal tissues below the Bio-PS during the process of charging–discharging (Fig. 6h, Supplementary Fig. S44).

## Discussion

The discovery of IMDs has demonstrated marked momentum for intervening and improving the lives of disabled people. Developing continuable power supply technologies in order to prolong the service life of IMDs without the need of renewing batteries by surgical assistance is preferred because it would be a better choice to satisfy the new generation of IMDs with higher energy consumption, such as electronic organs with the entire or partial functions of human organs. Though there is no clinical success to date, subcutaneous power supply by CPT has been recognized as a promising supplement to power IMDs. This line of research proposed a strategy of combining photothermal conversion with thermoelectric conversion, representing a new paradigm for subcutaneous power supply with the advantages of non-invasion, high output power, negligible biological damage, and deep tissue penetration. The output power of a packaged Bio-PS in the abdominal cavity of a rabbit reaches 20 mW under NIR-II light irradiation through the rabbit skin with a thickness of 8.5 mm. This value ranks as the highest output power among the existing examples of CPT that have been attempted or considered to be the subcutaneous power supply.

## Methods

### Fabrication of the Bio-PS

The PT layer coating on the TE generator was prepared by magnetron sputtered deposition technology. Typically, a Ni layer with a thickness of 500 nm was firstly deposited on the TE generator as the NIR-II light absorbing material (JCP200 high vacuum magnetron sputtering coating machine, Beijing Technol Science Co., Ltd.). Then a Ni/Al_2_O_3_ film was deposited on the top of the Ni layer by reactive RF magnetron co-sputtering which played the role of refraining the thermal radiation (LAB18 thin-film-deposition system, Kurt Co.). A SiO_2_ film was finally coated on the top of the Ni/Al_2_O_3_ layer, serving as an anti-reflective and enhanced photothermal layer (MSP-3200T, Beijing Chuangshiweina Technology Co.). Scanning electron microscope image of the layered PT film (Fig. 2a) was captured by Dimension icon (Bruker Co.).

TE generators (TEG-28706, Beijing Zhongfashidai Co., Ltd, China), Fresnel lens made of PMMA (purchased from Huaya Co., Ltd, China), and cooling fin made of copper (purchased from Xianweiguangneng Co., Ltd, China) were commercially available. The cup-like shell of Bio-PS, which was used to load bottom PCMs, cooling fin, TE generator, PT-layer, and upper layer from bottom to top in turn, was made from a photosensitive resin via 3D printing. The aluminum alloy base was prepared by 3D-printing technique (Supplementary Fig. S26f, g, purchased by Foshan Huilian Zhitong Painting Technology Co., Ltd., China).

Myristyl alcohol and carbon black were purchased from Heowens Co. Carbon black was oxidized by a mixture of concentrated sulfuric acid and nitric acid with a volume ratio of 1:3 so as to improve its dispersion in myristyl alcohol. The acidified carbon black was added into the myristyl alcohol at 50 °C with a fixed weight ratio of 5.0%, and a PCM with well-dispersed carbon black was obtained by ultrasonic dispersing technology (rpm: 8000 min^−1^).

### Characterization of PT layer’s photothermal conversion

NIR-II light was provided by a pumped solid-state laser (30 W, Ningbo Yuanming laser Co.). The ambient temperature was kept at 25 °C in the experiments. The photothermal conversion efficiency of the PT layer was calculated according to cycled temperature change curves (tested by RNO IR 384 camera) under on–off light irradiation (Supplementary Fig. S7a). The detailed calculation was provided in the Supplementary Discussion and Analysis.

### Characterization of the device

Both specific heat capacity and thermal conductivity of the materials presented in this work were important parameters for the measurement of thermal performance. The specific heat capacities were tested by differential scanning calorimetry (DSC822, Mettler Toledo Co.), and calibrated by Al_2_O_3_. Two heating cycles from 0 to 60 °C (specifically for PCM), or from 0 to 120 °C (for other materials) were recorded with a heating and cooling speed of 10 °C min^−1^, and all curves used in Supplementary Fig. S22 originated from the second cycle. Thermal gravity analysis (TGA) was recorded on a thermogravimetric analyzer (Nicolet 6700) at a ramp rate of 10 °C min^−1^ under nitrogen protection. The thermal conductivities of PCMs (Fig. 4c) or TE generator (Supplementary Fig. S20) over temperature were obtained by transient plane source method on Hot Disk TPS2500.

The UV–Vis–Infrared spectra of the PT layer and quartz separator were characterized on a diffuse reflection UV–Vis–NIR absorption spectrometer (UH4150, Hitachi Co.). The emissivity of the PT layer and quartz separator was tested by a Fourier transform infrared spectrometer (Vertex 70, Bruker Co.).

The internal resistance, open circuit voltage, and short circuit current of PTE converters under NIR-II irradiation with different power densities were recorded on a CHI 660 electrochemical workstation (Shanghai CH Instrument Co., Ltd, China). The sample interval is 0.1 s. In the temperature sensing tests, a thermostatic heater was used to control temperature (X200, Shenzhen Xinhaomai Electronic Technology Co., Ltd.).

All optical images and videos of noted experiments in the manuscript were captured by a Canon EOS 70D digital camera. All infrared images were recorded by RNO IR 384 camera.

### Circuit design of involved electron devices

As depicted in Supplementary Fig. S32, a special step-up transformer was used to enlarge the output voltage of Bio-PS under a given light power. In the circuit diagram, the key chip was BQ25504 which was purchased from Dalian Kaicheng design Co. Ltd, China. Practically, such a special step-up transformer could increase the output voltage of Bio-PS to 5.3 V steadily. In this voltage transformation process, the ratio of energy loss was estimated at <20%. The implanted wireless camera in the vivo experiment was composed of a GC0308 camera (640 × 480 pixel) driven by stm32l4 MCU, a nrf52832 versatile Bluetooth, a magnetic switch (Normally open type), and a polymer lithium battery (5 V, 40 mAh). The circuit diagram was specifically demonstrated in Supplementary Fig. S41, and its maximum consumption was over 100 mW, and its average energy consumption of 55.65 mW (Supplementary Fig. S42). The frame of this wireless camera was 0.5 fps. The video captured by the wireless camera could be sent timely from the body to the mobile phone via Bluetooth.

### Biological assessments of Bio-PS performance in vitro

Bio-PS covered by biological tissue was placed on a thermostatic heater with a temperature of 37 °C and used to provide direct or indirect power supply for electrical appliances under NIR-II irradiation with LPD of 1.0 W m^−2^. Three kinds of biological tissues were used to identify the capability of subcutaneous power supply by NIR-II light, including 3.5 mm-thickness pigskin, 20 mm-thickness pork, and the tissue of rabbit’s abdomen (skin and tissue together, around 8.5 mm in thickness). The ambient temperature was 25 °C. The direct power supply referred to the electrical appliances directly connected to Bio-PS, and the indirect power supply referred to the electrical appliances connected with a battery or capacitor which was charged by Bio-PS. The charging processes of the indirect power supply were under on–off light irradiation with 10 min intervals, which significantly reduced the risk of tissue burning. The electrical appliances, including LEDs, fan, battery, supercapacitor, and camera with WIFI (H8-5, Honggu Co., Ltd) were commercially supplied by Beijing Zhongfashidai Co., Ltd.

### Biological assessments of Bio-PS performance in vivo

The study was authorized by the Tab of Animal Experimental Ethical Inspection Animal Experimental Center of Nongnong (Beijing) Life Science & Technology Company (authorization number: 202201209, Beijing, China). And they were performed in accordance with the Regulations for the Administration of Affairs Concerning Experimental Animals approved by the State Council of the People’s Republic of China. The rabbits were New Zealand white rabbits (2.0−2.5 kg), and purchased from Beijing NongNong Bio-Technology Co. Ltd. According to the American National Standard for Safe Use of Lasers, ANSI Z136.1-2007, the continuous NIR-II light irradiation time in the vivo experiment did not exceed 10 min and the LPD of NIR-II light is 1 W cm^−2^.

### Measurements of output power and temperature change

The Bio-PS was implanted into the abdominal cavity of rabbits. The output open-circuit voltage and short-circuit current were measured on a CHI 660 electrochemical workstation. A comparison of Bio-PS with some other power supply devices for the applications of IMDs was supplied in Supplementary Table 3. Notably, Bio-PS was able to generate an output power of around 20 mW, exhibiting great advantages of high power generation and deep tissue penetration.

### Direct power supply for a pacemaker

A pacemaker for animals (purchased from the information center of First Hospital of Hebei Medical University) and Bio-PS were implanted into a rabbit’s abdominal cavity. The lead pacemaker was anchored on the cardiac surface by suture. After the implanting of the pacemaker and Bio-PS, the rabbit abdomen was exposed to NIR-II light irradiation, and the electrocardiogram of the rabbit was monitored at the same time (Fig. 6d). In principle, the pacemaker with a frequency of 350 Hz at activated status could speed up the heart rate of rabbit. Therefore, the change in the rabbit’s electrocardiogram under NIR-II light irradiation would be an indicator of the successful power supply by Bio-PS work.

### Indirect power supply for an implanted camera

After the rabbit was anesthetized, Bio-PS was implanted into the abdominal cavity of the rabbit, and a wireless endoscope with a size of 1.5 × 1.5 × 3 cm^3^ was implanted into a different place of the rabbit. Supplementary Fig. S41 illustrated the circuit diagram of how the wireless endoscope was connected with Bio-PS. After the closure of the surgical wound, the wireless endoscope was charged by Bio-PS under NIR-II light irradiation for 2 h (Supplementary Fig. S43). The camera was then activated once the magnetic switch in the circuit was turned on and in vivo images were sent to a mobile phone via Bluetooth.

### Safety of Bio-PS

The Bio-PS was left in the rabbit’s body for one month to evaluate its biological safety. As shown in Fig. 6h and Movie S9, the rabbit resumed eating and exercising on the next day after surgery and survived well during the one-month observation without the appearance of nursing complications. The biological tissues closely contacted with the upper and lower surfaces of the Bio-PS device and light-irradiated skin were taken for H&E staining (Figs. 5f, 6h, and Supplementary Fig. S44, Beijing Boaosaien Technology Co., Ltd.). The H&E staining results revealed that there was no evident inflammation for the tissues surrounding the Bio-PS device, and the skin cuticle where NIR-II light was applied was preserved very well. Besides, the typical characteristic of laser burning, including the damage of the corium layer and basophilic changes of the connective tissue layer, were not observed in the studies of HE staining. The images of the standard H&E staining are given in Figs. 5f, 6h, and Supplementary Fig. S44 are representative results, which are similar to the results of the other independent experiments.

### Statistics and reproducibility

All analyses were performed in OriginPro 2021 and Microsoft Excel 2020. All the simulations were implemented using the Ansys Fluent software (version number: 19.0). No data were excluded from the analyses.

### Reporting summary

Further information on research design is available in the Nature Research Reporting Summary linked to this article.

## Supplementary information

Supplementary Information

Description of Additional Supplementary Files

Supplementary Movie S1

Supplementary Movie S2

Supplementary Movie S3

Supplementary Movie S4

Supplementary Movie S5

Supplementary Movie S6

Supplementary Movie S7

Supplementary Movie S8

Supplementary Movie S9

Reporting Summary

## Data Availability

The authors declare that all experimental data and relevant analysis of this work are included in the main manuscript and its Supplementary Information, and available from the corresponding author upon request.
