# Supplementary material for: Subcutaneous power supply by NIR-II light
Source: Nat Commun. 2022 Nov 3;13:6596. doi: 10.1038/s41467-022-34047-5 (PMC9633840; doi:10.1038/s41467-022-34047-5)
Supplement: Supplementary file 1 — Supplementary Information [file 41467_2022_34047_MOESM1_ESM.docx]

**Supporting information**

**Subcutaneous Power Supply by NIR-II Light**

Shanzhi Lyu^1,2^, Yonglin He^1^, Xinglei Tao^1^, Yuge Yao^2^, Xiangyi Huang^1^, Yingchao Ma^1^, Zhimin Peng^2^, Yanjun Ding^2^, Yapei Wang^1^*

^1^ Key Laboratory of Advanced Light Conversion Materials and Biophotonics, Department of Chemistry, Renmin University of China, Beijing, 100872, China

^2^ Department of Energy and Power Engineering, Tsinghua University, Beijing, 100084, China

**1. Supplementary Discussion and Analysis** 4-10

1.1 Analysis of horizontal heat transfer involved in PTE converters 4

1.2 Non-steady-state heat transfer model 5-7

1.3 Analysis of radiation heat transfer 8-9

1.4 Calculation of photothermal conversion efficiency 10

**2. Simulation study and analysis of result** 11-17

2.1 For non-steady-state heat transfer 11-12

2.2 For upper layer 13-15

2.3 For PCM and bottom layer 16-17

**3. Supplementary Tables and Figures**  18-47

**4. References for Supporting Information**  48-50

Other Supplementary Information files for this manuscript include the following: Movie S1 to S9

**Content of Supplementary Table and Figure**

| **Section** | **Title** | **Figure and table** | **Page numbers** | |
| --- | --- | --- | --- | --- |
| **1** | Heat transfer model | Table 1, 2 and Figure S1-S5 | 18-19 and 22-24 |  |
| **2** | Photothermal Characterization of PT layer | Figure S6-S10 | 24-26 |  |
| **3** | Simulation and Characterization of Upper layer | Figure S11-S18 | 27-31 |  |
| **4** | Characterization of TE generator | Figure S19 S20 | 32 |  |
| **5** | Simulation and Characterization of PCM and cooling Fin | Figure S21-S27 | 33--36 |  |
| **6** | Electronic and safety characterization of Bio-PS | Figure S28-S30 | 37, 38 |  |
| **7** | In vitro assessment of Bio-PS | Figure S31-S37 | 38-42 |  |
| **8** | In vivo assessment of Bio-PS | Table 3 and Figure S38-S44 | 20-21 and 43-47 |  |

1. **Supplementary Discussion and Analysis**

**1.1 Analysis of horizontal heat transfer involved in PTE converters.**

The heat flux of the free convection of the horizontal heat transfer is:

$q=H*\Delta t=\frac{A_{1-3}*Nu*\lambda*\left( t_{\mathrm{PT}}-t_{\mathrm{side}} \right)}{d_{13}}$ (eq. S1)

where *H* is the free convection coefficient, *λ* is the air heat conductivity, *t*_PT_ and *t*_side_ are the temperature of two surfaces, *d*_13_ is the distance between two surfaces and *Nu* is the Nusselt number in the longitudinal and horizontal directions, which could represent the intensity of free convection^1^.

$Nu=f(Pr,Gr*\cos\theta)$ (eq. S2)

where *Gr* and Pr are the Grashof number and Prandtl number, *θ* is the angle between the PT layer and the sidewall^2-3^. For the heat flux between the PT layer and sidewall, the PT layer is perpendicular to the sidewall, thus *θ is* 90^o^. Accordingly, cos*θ is* 0 and the heat flux between the PT layer and sidewall is much less than the heat flux between the PT layer and top layer. Thus, there is little free convection in the horizontal direction and it could be neglected in comparison with the longitudinal direction. The experiment results of the former manuscripts also confirmed this explanation ^4-5^.

The heat radiation could be considered as:

$\Phi=\varepsilon*A_{1}*X*\sigma*\left( t_{PT}^{4}-t_{side}^{4} \right)$ (eq. S3)

where *Φ* is the heat radiations between the PT layer and sidewall.*ε* are the emissivities between the PT layer and sidewall as well as between the PT layer and the top surface. *A*_1_, *A*_3_ are the areas of the PT layer and the sidewall, respectively. *X* is the radiation shape factor:

$X=\frac{1}{A_{1}}\int_{A_{1}} \int_{A_{3}} \frac{\cos\theta_{1}\cos\theta_{3}dA_{1}dA_{3}}{\pi r^{2}}$ (eq. S4)

where 𝜃 _1_ and 𝜃_3_ are the angles of the d*A*_1_ and d*A*_3_. In the case that the thickness of the upper layer is 6 mm, *d*_13_ is 2 mm, and the length of the device is 60 mm, querying the corresponding data table^6^ can find that *X* =0.16. Therefore, *X* is a negligible value in comparison, and so is *Φ* remarkably inferior to total heat flux_._

**1.2 The non-steady-state heat transfer model involved in PTE converters.**

Referring to the model as presented in Scheme 1c, a coordinate system was established with the center of PT layer as the origin (blue point), and the downward direction was defined as the positive axis. It could be considered as a one dimensional non-steady-state heat transfer model, which possessed a second boundary condition and a third boundary condition on the top surface, and a third boundary condition on the bottom surface. Those symbols in the heat transfer model were specifically explained in Supplementary Table 2. At the time of *τ*, Eq. (1) was transformed as:

$P_{PT}-P_{loss}\left( \tau\right)=P_{trans}^{*}\left( \tau\right)$ (eq. S5)

where, *P*_loss_(*τ*) and $P_{trans}^{*}\left( \tau\right)$ represent the time-dependent powers. $P_{trans}^{*}\left( \tau\right)$ is divided into the consumption in the generation of thermoelectric power ($P_{\mathrm{op}}\left( \tau\right)$), the thermal storage by the TE generator itself ($P_{\mathrm{TEG}}\left( \tau\right)$), and the rest thermal energy arriving at the bottom surface of TE generator ($P_{\mathrm{bottom}}\left( \tau\right)$):

$P_{trans}^{*}\left( \tau\right)=P_{\mathrm{op}}\left( \tau\right)+P_{\mathrm{bottom}}\left( \tau\right)+P_{\mathrm{TEG}}\left( \tau\right)$ (eq. S6)

Here *P*_op_(*τ*) is described by:

$P_{op}(\tau)=\frac{S^{2}\left( t \right){[t_{1}\left( \tau\right)-t_{2}\left( \tau\right)]}^{2}}{R(t)}$ (eq. S7)

Similarly, *P*_loss_(*τ*) and *P*_bottom_(*τ*) are determined as $P_{\mathrm{loss}}\left( \tau\right)=H_{\mathrm{upper}}[t_{1}\left( \tau\right)-t_{0}\left( \tau\right)]$, and $P_{\mathrm{bottom}}\left( \tau\right)=H_{\mathrm{bottom}}[t_{2}\left( \tau\right)-t_{3}\left( \tau\right)]$. The rest thermal energy transferred to the medium under TE generator is termed as *P*_bottom_(*τ*), ^7^

$\left| \begin{aligned} \frac{\partial^{2}\theta}{\partial x^{2}}=\frac{1}{\alpha}\frac{\partial\theta}{\partial\tau} \alpha=\frac{\kappa_{\mathrm{TEG}}}{{\rho_{\mathrm{TEG}}*Cp}_{\mathrm{TEG}}} \\ {-\frac{\partial\theta}{\partial x}\lambda|}_{x=0}=P_{\mathrm{PT}}-H_{\mathrm{upper}}\theta\\ {\frac{\partial\theta}{\partial x}\lambda|}_{x=L}={-H}_{\mathrm{bottom}}\theta\\ \tau=0, \theta=0 \end{aligned} \right.$ (eq. S8)

*α* is the thermal diffusivity, and *θ* is excess temperature as *θ*=*t*-*t*_body_, which can be also indicated as:

$\theta=M\tau+\theta_{1}+\theta_{2}$ (eq. S9)

The variation of inhomogeneous steady state is

$\left| \begin{aligned} \frac{\partial^{2}\theta_{1}}{\partial x^{2}}=\frac{A}{\alpha} \\ {-\frac{\partial\theta_{1}}{\partial x}\lambda|}_{x=0}=P_{\mathrm{PT}}-H_{\mathrm{upper}}\theta_{1} \\ {\frac{\partial\theta_{1}}{\partial x}\lambda|}_{x=L}=-H_{\mathrm{bottom}}\theta_{1} \end{aligned} \right.$ (eq. S10)

$$\theta_{1}=C_{1}x^{2}+C_{2}x+C_{3}$$

And the variation of homogeneous non-steady state is

$\left| \begin{aligned} \frac{\partial^{2}\theta_{2}}{\partial x^{2}}=\frac{1}{\alpha}\frac{\partial\theta_{2}}{\partial\tau} \\ {-\frac{\partial\theta_{2}}{\partial x}\lambda|}_{x=0}=P_{\mathrm{PT}}-H_{\mathrm{upper}}\theta_{2} \\ {\frac{\partial\theta_{2}}{\partial x}\lambda|}_{x=L}=-H_{\mathrm{bottom}}\theta_{2} \end{aligned} \right.$ (eq. S11)

$$\theta_{2}=\sum_{m=1}^{\infty} \left\{ e^{\alpha\beta_{m}^{2}\tau}\frac{1}{N^{2}\beta_{m}}\left[ \beta_{m}\cos\left( \beta_{m}x \right)+H_{upper}sin(\beta_{m}x) \right]\times\int_{0}^{L} (-)\left[ \beta_{m}\cos\left( \beta_{m}x \right)+H_{upper}sin(\beta_{m}x) \right]dx \right\}$$

Here, *β*_m_ is a dimensionless criterion, tan*β*_m_L= *β*_m_*(*H*_upper_+*H*_bottom_)/(*β*_m_-*H*_upper_**H*_bottom_), m=1,2,3… and so on. When Fourier number Fo>0.2, the m could be approximated to defined as 1. And:

$\frac{1}{N^{2}}=\frac{2}{\left( \beta_{m}^{2}+H_{upper}^{2} \right)\left( L+\frac{H_{bottom}^{2}}{\beta_{m}^{2}+H_{bottom}^{2}} \right)+H_{upper}}$ (eq. S12)

By resolving the above equation group, it is concluded that the temperature difference between *t*_1_(*τ*) and *t*_2_(τ) is positively correlated with *P*_bottom_(*τ*), *P*_PT-λ_, and *H*_bootom_ while negatively influenced by *P*_loss_(*τ*) and *H*_upper_. This conclusion is constant with the steady-state heat transfer model in the main text. Furthermore, it is predicted that the temperature difference between *t*_1_(*τ*) and *t*_2_(τ) does not keep increasing during continuous NIR-II light irradiation when *H*_upper_> *H*_bootom_. Its variation trend will be shown as increasing to maximum value first, then decreasing.

More analysis is supplied and discussed in the results and discussion of the simulation.

**1.3 Reduction of radiation heat transfer by quartz plate**

The quartz plate was used as a radiation shield to reduce radiation heat transfer, with which some of the radiation heat transfer could be reduced due to the reflection of the quartz plate, and the rest radiation heat transfer could be considered as:

${P_{rad-Qz}=\Phi}_{PT-Lens}+P_{transmiss}$ (eq. S13)

Here, *Φ*_PT-Len_ represents the indirect radiation heat transfer of the PT layer via the quartz plate, and *P*_transmiss_ represents the direct radiation heat transfer of the PT layer through the quartz plate. The transmission of the quartz plate is around 0.05 (Supplementary Fig. S15)

The temperature difference between two sides of the quartz separator was negligible due to its ignorable thickness. As for the upper layer of the quartz separator, the radiative heat transfer could be considered as:

$\Phi_{PT-Lens}=\Phi_{PT-Qz}=\Phi_{Qz-Lens}$ (eq. S14)

Here, *Φ*_PT-Qz_, and *Φ*_Qz-Lens_ represent the radiant heat transfer between the PT layer and the quartz separator, and between the quartz separator and the lens, respectively. According to eq. S14, the heat radiation of two parallel plates is in a closed space, the *Φ*_PT-Lens_ could be obtained as:

$\Phi_{PT-Lens}=\frac{1}{\frac{1}{\varepsilon_{PT}}+\frac{1}{\varepsilon_{Lens}}-1}(E_{b1}-E_{b2})$ (eq. S15)

Similarly, the *Φ*_PT-Qz_, and *Φ*_Qz-Lens_ are:

$\Phi_{PT-Qz}=\frac{1}{\frac{1}{\varepsilon_{PT}}+\frac{1}{\varepsilon_{Qz}}-1}(E_{b1}-E_{b3})$ (eq. S16)

$\Phi_{Qz-Lens}=\frac{1}{\frac{1}{\varepsilon_{Qz}}+\frac{1}{\varepsilon_{Lens}}-1}(E_{b3}-E_{b2})$ (eq. S17)

Here, *ε*_PT_, *ε*_Qz_, and *ε*_Lens_ are the emissivity of the PT layer, the quartz separator and the lens, which are 0.35, 0.8, and 0.95 on average, respectively. Taking the data into the eq. S17, it could be calculated that the reduction of radiative heat transfer by quartz plate is around 55%.

**1.4 Calculation of photothermal conversion efficiency of PT layer**

The photothermal conversion efficiency of PT layer (*η*_PT_) could be calculated according to eq. S18.

$\eta_{PT}=\frac{h^{'}A\Delta t_{max}}{P_{light}}$ (eq. S18)

Here, Δ*t*_max_ is the temperature difference between the maximum steady-state temperature of the photothermal materials and ambient temperature, *A* is the surface area of photothermal materials, *h’* is the equivalent heat-transfer coefficient, and *P*_light_ is the power of NIR-II light (*P*_light_ =1 W). In order to get *h’A*, eq. S19 based on the conservation of energy in the cooling process was elucidated^8^.

$mC_{p}\frac{dt}{d\tau}=-h'A(t-t_{\mathrm{begin}})$ (eq. S19)

Here, *mC*_p_ is the sum heat capacity of the copper sheet because the heat capacity of the PT layer is small enough that could be negligible. So eq. S19 could be simplified as:

$\tau=\frac{mC_{p}}{h^{'}A}\times ln\theta$ (eq. S20)

ln*θ* is defined as *t*/*t*_begin_. However, it's hard to measure the *C*_p_ of TE generator accurately. Therefore, the PT layer was coated on the copper sheet. Their total weight (*m*) was 1.2816 g and *C*_p_ was obtained as 0.39 J g^-1^ ^o^C^-1^. Therefore, *h’A* was calculated by the linear correlation between the cooling period t and –ln*θ* (Supplementary Fig. S7b). Thus, the PT layer’s *η*_PT_ was specified by solving eq. S18-S20, which was calculated as 93.98%.

**2. Simulation settings and analysis of results**

In order to evaluate the impact of those parameters used in the non-steady-state heat transfer model, the optimized design of upper layer, the performance of PCMs with or without carbon black, three unsteady-state heat transfer models had been established, and their temperature distributions were predicted^9^.

The initial temperature of the whole simulated object was 37 ^o^C. The properties of the materials used in the simulations, including specific heat capacity, thermal conductivity, and transmittance, were consistent with the above experimental tests or previous literature. The process of all simulations lasted 600 s.

**2.1 For unsteady-state heat transfer**

In this simulation, the impacts of the parameters of *H*_1_, *H*_2_, *t*_3_, and *P*_PT_ on the temperature difference of TE generator (*t*_1_- *t*_2_) were evaluated. The model was proposed as a TE generator, whose top surface transferred heat through the air layer, and the bottom surface transferred heat through the bottom layer. The length, width, and height of the TE generator were 40 mm, 40 mm, and 11.4 mm, respectively. The gird independence had been tested and a total of 19200 cells were adopted. The temperature of tissue was considered as a constant of 37 ^o^C.

Firstly, *H*_1_ and *t*_3_ were regarded as constants of 10 W m^-2^ ^o^C^-1^, 37^o^C, respectively. The (*t*_1_- *t*_2_) changed under the influence of *P*_PT_ and *H*_2_. The curve of (*t*_1_- *t*_2_) over *H*_2_ could be described as Supplementary Fig. S2, and *P*_PT_ were 3.0 W, 4.0 W, 5.0 W, or 6.0 W corresponding to Supplementary Fig. S2a-d, respectively. It was noted that increasing *P*_PT_ was beneficial to enlarge (*t*_1_- *t*_2_) under the same boundary condition. And when *H*_1_, *t*_3_, *P*_PT_ were fixed, the change of temperature difference (*t*_1_- *t*_2_) over *H*_2_ increased first and then remained at a certain level. Specifically, (*t*_1_- *t*_2_) reached the maximum with *H*_2_ of 100 W m^-2^ ^o^C^-1^.

Moreover, when *P*_PT_, *H*_2_, *t*_3_, were regarded as constants of 5 W, 10 W m^-2^ ^o^C^-1^, 37^o^C, respectively. The curve of (*t*_1_- *t*_2_) over time could be described as Supplementary Fig. S3. When *H*_1_ $\leq$ *H*_2_, (*t*_1_- *t*_2_) increased constantly over time. However, if *H*_1_ > *H*_2_, a peak of (*t*_1_- *t*_2_) would reach quickly. Subsequently, the curve would fall immediately and reach a plateau finally.

Besides, when *P*_PT_, *H*_1_, *H*_2_, were regarded as a constant of 3.0 W, 10.0 W m^-2^ ^o^C^-1^, 100 W m^-2^ ^o^C^-1^, respectively, the curve of (*t*_1_- *t*_2_) over *t*_3_ was shown in Supplementary Fig. S4. It could be found that (*t*_1_- *t*_2_) decreased as *t*_3_ increased. Based on the above simulation results, it was concluded that *t*_1_- *t*_2_ was enlarged as increasing *P*_PT_ and *H*_2_ or reducing *H*_1_ and *t*_3_, which was fully consistent with the steady-state heat transfer model (Discussion of the main text), and unsteady-state heat transfer model (Supplementary Discussion 2.1).

**2.2 For the upper layer**

The aim of optimizing the upper layer was to reduce *P*_loss_, increase (*t*_1_ - *t*_2_), and keep *t*_0_ at a low level to ensure the safety of the PTE device. The time-scale temperature of the PTE’s top surface under different conditions, including varied thicknesses of air layer with or without a quartz separator were obtained. In this simulation, the PTE device could be considered as a cylinder with a radius of 30 mm, which was composed of an upper layer of air, a PT layer, and a Fresnel lens and surrounded biological tissue. Two models were established with or without using a quartz separator with a thickness of 1 mm to separate the upper air layer. The length, width, and height of the PT layer were 40 mm, 40 mm, and 11.4 mm, respectively. A total of 144039 cells were adopted for the upper layer (see Supplementary Fig. S14). In this simulation, the convective heat transfer coefficient of the tissue surface was regarded as a constant of 10 W m^-2^ ^o^C^-1^, and the ambient temperature was a constant of 25 ^o^C. The temperature change of PT layer was the same as the results of the photothermal conversion experiments (Supplementary Fig. S10).

The LPD was chosen as 0.3 W m^-2^. The thickness of the upper layer (air and quartz separator together, corresponding to *d*) was attempted as 1.6 mm, 2.6 mm, 3.6 mm, 4.6 mm, 5.6 mm, 6.6 mm, 7.6 mm, 8.6 mm, 9.6 mm, 10.6 mm, 11.6 mm and 12.6 mm, and the corresponding temperature distributions were predicted as shown in Fig. 3d and. Supplementary Fig S12-13.

According to the above simulation results, the curves of *t*_0_ over *d* with or without quartz separator were summarized and compared in Fig. 3c. In the model without quartz separator, the trend of Δ*t*_0_ decreased dramatically as *d* increased and then rose before and after a critical thickness of the air layer. While, in the model with quartz separator, the trend of Δ*t*_0_ decreased as *d* increased all the time. However, when the thickness was 1.6 mm or 2.6 mm, the heat convection was low enough to be negligible, and the heat conduction held a dominant position in heat transfer (similar as Supplementary Fig. S11a). In such a condition, quartz separator could intensify heat transfer and further cause the increase of *t*_0_. These observations were consistent with the trend of *h*_1_ in the discussion and analysis of the main text.

Moreover, only when the upper layer’s thickness in the model with quartz separator was not less than 8.8 mm, Δ*t*_0_ could be less than the critical temperature of 5 ^o^C for the tissue safety. Therefore, in this work, the upper layer’s thickness was chosen as 8.8 mm, and a quartz separator was placed in the middle of the upper layer.

The above simulation results offered evidence to confirm the tissue safety, validity, and rationality of the upper layer for the design of packaged PTE devices.

**2.3 For PCM and bottom layer**

According to the analysis of heat transfer models, *t*_3_ and *t*_4_ were influenced by the bottom layer. The thermal conductivity of PCM composed of myristyl alcohol would increase with the addition of carbon black, but its specific heat capacity was decreased at the same time. In this section, the model could be considered as a polymer cup with a square opening at its top, which was sealed by a copper square lid. The cup radius was 30 mm, and the length of the square opening and copper lid were both 40 mm. The thicknesses of the cup wall and the lid were 0.5 mm. The rest space of the cup was filled with PCM or carbon black-modified PCM. There was biological tissue surrounding the cup. A total of 191661 cells were adopted for this part (see Supplementary Fig. S25).

The variable heat flow was placed on the top surface of the lid. It gradually increased from 0 W, and at the time of 200s, it reached the maximum of 3 W and then remained unchanged. The time-scale temperature distributions of the model with the use of PCM or the carbon black-modified PCM device were obtained (Fig. 4d, Supplementary Fig. S23-24). The simulation results demonstrated that *t*_3_ of the device loaded with carbon black-modified PCM was lower than the device with PCM only, while *t*_4_ of them were similar.

The temperature changes over PCM height (L) were supplied in Fig. 4d. According to the characterizations of PCMs with or without carbon black, it was revealed that the melting temperatures of PCM and carbon black-modified PCM were around 40 and 44 ^o^C, respectively. In comparison, the temperature distribution of the device loaded with the carbon black-modified PCM was more uniform. The simulation and experiments as presented in Fig. 4e proved that the carbon black facilitated better utilization of the heat capacity of PCMs.

Similarly, the effect of the cooling fin was also been testified by FEM. As predicted in Supplementary Fig. S27, *t*_2_ further decreased to 45 ^o^C under irradiation of 0.3W cm^-2^ when cooling fin and PCM were used at the same time.

These simulation studies as stated above were also supported by experimental investigations (Fig. 4e).

**3. Supplementary Table and Figure**

**Supplementary Table 1**. Symbols appeared in the steady-state heat transfer model

| **Symbol** | **Physical meanings** | **Symbol** | **Physical meanings** |
| --- | --- | --- | --- |
| *P*_PT_ | Heat power by photothermal conversion | *P*_loss_ | Heat loss power of PT layer |
| *P*_trans_ | Power of heat transporting through the PTE device | *P*_op_ | Output power of Bio-PS |
| *P*_light_ | NIR-II light’s power | *P*_bottom_ | Heat transfer power between TE generator and the lower encapsulating |
| *P*_rad_ | Radiation heat loss power of PT layer | *P*_conv_ | Convective heat transfer power via upper layer |
| *t*_0_ | Temperature at the total device’s top surface | *t*_1_ | PT layer’s temperature |
| *t*_2_ | Temperature at the TE generator’s bottom surface | *t*_3_ | Temperature at the top surface of bottom layer |
| *t*_4_ | Temperature at the total device’s bottom surface | *D* | Thickness of TE generator |
| *S*(*t*) | Seebeck coefficient of TE generator | *R*(*t*) | The device’s resistance |
| *T* | Tissue’s transmittance | *η*_PT_ | Efficiency of photothermal conversion |
| *λ* | Thermal conductivity of TE generator | *h*_1_ | Convective heat  transfer coefficients of upper layer |
| *H*_1_ | Heat loss coefficient | *A*_1_ | Area of PT layer |
| *H*_2_ | Heat transfer coefficients between the TE generator’s bottom surface and the lower encapsulating | *A*_2_ | Heat transfer area correspond to *H*_2_ |
| *A*_0_ | Area of the tissue in contact with the top surface of the PTE converter | *σ* | Stefan-Boltzmann constant |
| *ε*_1_ | PT layer’s emissivity | *ε*_2_ | Tissue’s emissivity |
| *d* | Thickness of air layer | *L* | Thickness of PCM layer |

**Supplementary Table 2**. Other Symbols in the non-steady-state heat transfer model of Supplementary Discussion.

| **Symbol** | **Physical meanings** | | **Symbol** | **Physical meanings** | |
| --- | --- | --- | --- | --- | --- |
| *τ* | | Irradiation time | *P*_loss_(τ) | | Heat loss power from device bottom surface to body at τ |
| $P_{trans}^{*}$(τ) | | Heat transfer power from x=0 to x=L | *P*_bottom_(τ) | | *P*_bottom_ at τ |
| *P*_op_(τ) | | Maximum output power at τ | *P*_TEG_(τ) | | Power of absorbing heat of TEG at τ |
| *t*_0_(τ) | | *t*_0_ at τ | *t*_1_(τ) | | *t*_1_ at τ |
| *t*_2_(τ) | | *t*_2_ at τ | *t*_3_(τ) | | *t*_3_ at τ |
| *H*_upper_ | | Equivalent convection heat transfer coefficient of upper layer | *H*_bottom_ | | Equivalent convection heat transfer coefficient of bottom layer |
| *β*_m_ | | Dimensionless criterion | Fo | | Fourier number |
| *α* | | Thermal diffusivity | *θ* | | Excess temperature |
| *M* | | Coefficient of factor of *τ* | *N*^2^ | | Dimensionless criterion of homogeneous non-steady state |

**Supplementary Table 3**. Comparison of different types of power supply devices for IMDs.

| **Ref.** | **Light Source** | **Method** | **Model** | **Depth(mm)** | **Output power(μW)** |
| --- | --- | --- | --- | --- | --- |
| 10 | Sun light | PV | Porcine(vitro) | 0/2/4 | 122/44.9/25 |
| 11 | Wireless (1.6Ghz) | RF | Rabbit(vivo) | 50/100 | 195/10 |
| 12 | Sun light | PV | Rat(vivo) | 0.5^*^ | 647 |
| 13 | Wireless (88Mhz) | RF | Water | 20 | 700 |
| 14 | Sun light | PV | Porcine(vitro) | 3 | 5500 |
| 15 | Sun light | PV | Pig(vivo) | 2.8 | 3981 |
| 16 | Sun light | PV | Rat(vivo) | 0.5^*^ | 8.2 |
| 17 | NIR-II | PV | Porcine(vitro) | 1/6 | 280/20 |
| 18 | NIR-I | PV | Porcine(vitro) | 4/6 | 12.24/8.04 |
| 19 | NIR-I | PV | None | 0 | 1.65 |
| 20 | Sun light | PV | Porcine(vitro) | 4^*^ | 0.12 |
| 21 | NIR-II | PV | Porcine(vitro) | 1^*^ | 3440 |
| 22 | bioenergy | Piezo | Pig(vivo) | -- | 300 |
| 23 | bioenergy | . Biofuel Cells | Rat(vivo) | -- | 135 |
| 24 | bioenergy | ionic migration | Nono | -- | 50 |
| 25 | bioenergy | . Biofuel Cells | Rat inner ear (vivo) | -- | 0.0063 |
| 26 | bioenergy | Piezo | Pig | -- | 30.972 |
| Bio-PS | NIR-II | PTE | Rabbit(vivo)/ Porcine(vitro) | 3.5/8.5/20 | 190000/20000/6000 |

^*^ represents the estimated implanted depth that was not provide the value by the related paper.

-- represents the implanted depth which is not major concern in those related papers.

**
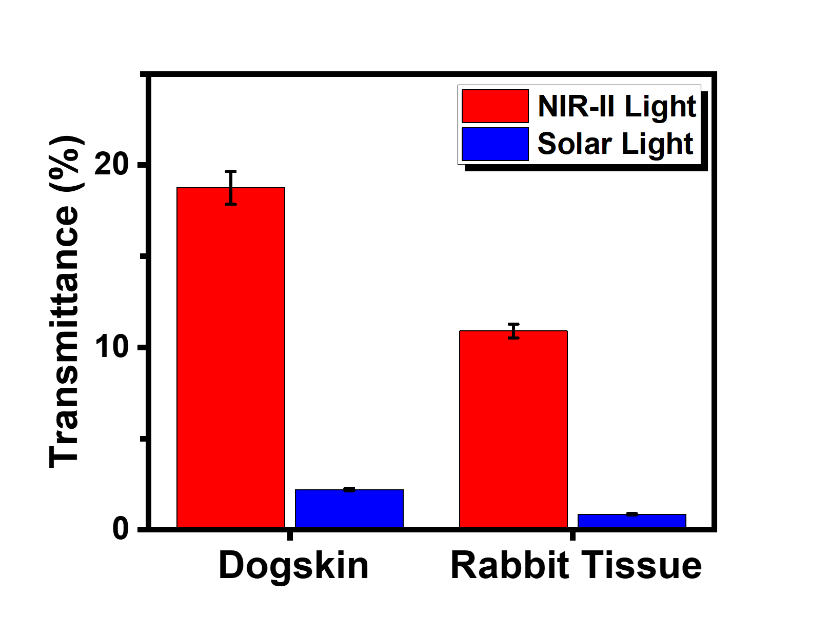
**

**Supplementary Fig. 1 | The transmittance of NIR-II light (red) and solar light (blue) through dogskin or rabbit tissue.** The values of transmittance are 18.75±0.90%, 2.20±0.06%, 10.89±0.38%, and 0.85±0.04%, respectively. (n=6)

**
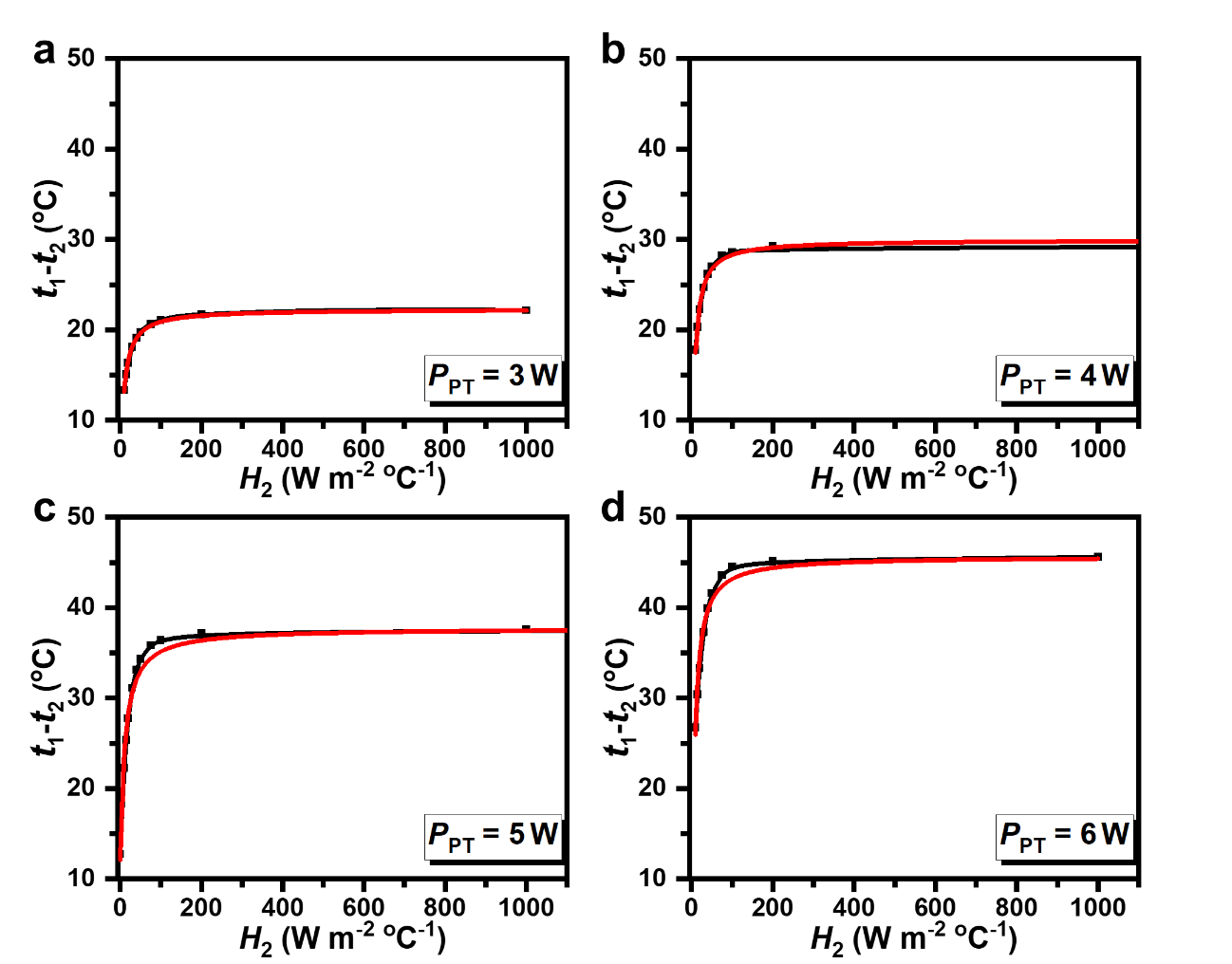
**

**Supplementary Fig. 2 | The simulated *t*_1_-*t*_2_ over different *H*_2_ after NIR-II light irradiation for 10 min. a-d.** The LPD of incident light was 3.0 W, 4.0 W, 5.0 W, 6.0 W, respectively. The initial temperature in the simulation model was kept as 37^o^C.


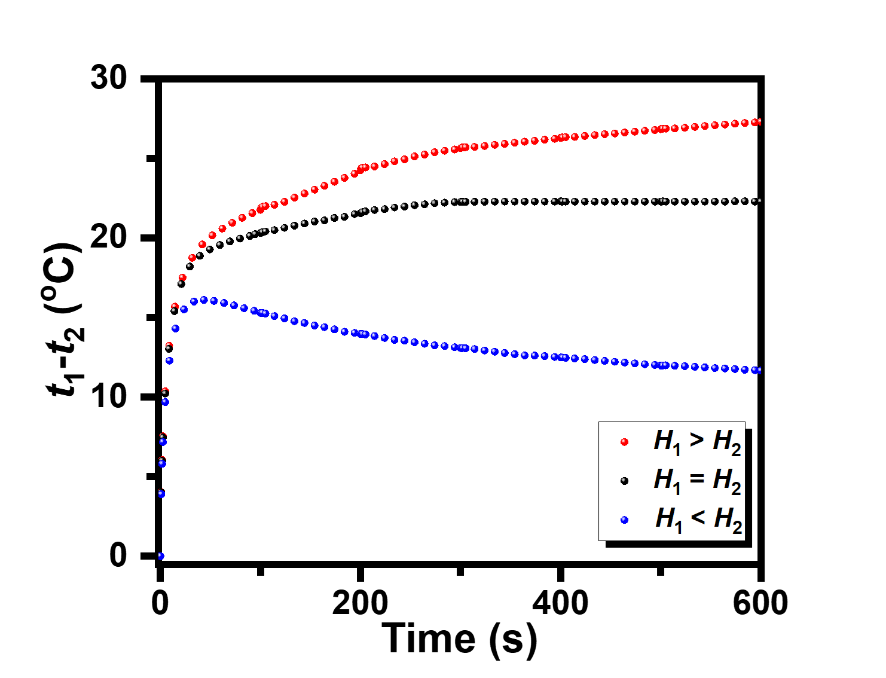


**Supplementary Fig. 3 | The simulated time-dependent *t*_1_-*t*_2_.** The red, black, and blue curve represented *H*_1_ > *H*_2_, *H*_1_ = *H*_2_, and *H*_1_ < *H*_2_, respectively. The initial temperature in the simulation model was 37^o^C.


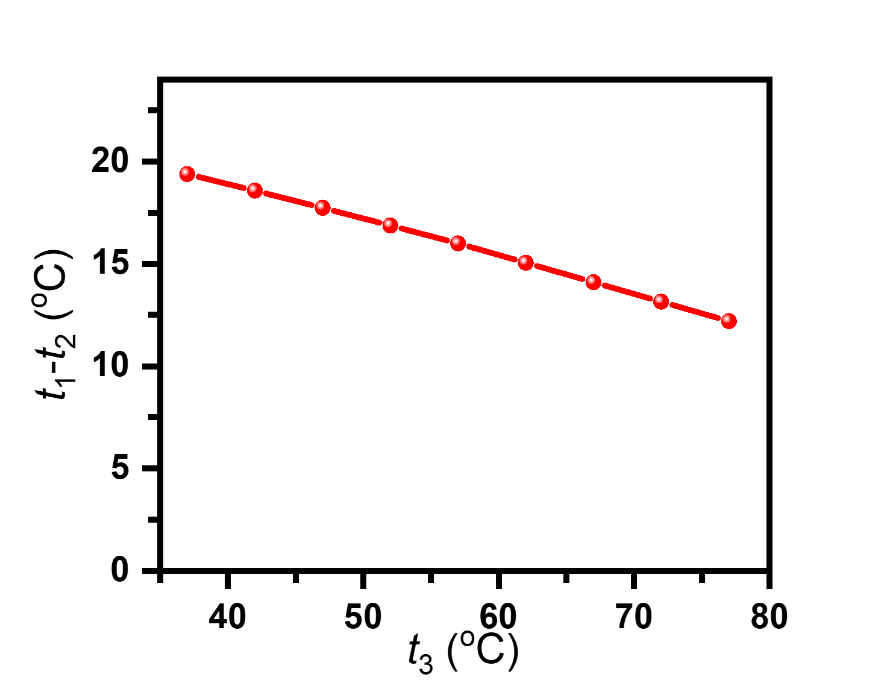


**Supplementary Fig. 4 | The simulated *t*_1_-*t*_2_ as function of *t*_3_ after NIR-II light irradiation for 10 min.** The *H*_1_, *H*_2_ and heat flux were 10 W m^-2^ ^o^C^-1^, 200 W m^-2^ ^o^C^-1^ and 3.0 W, respectively. The initial temperature in the simulation model was 37^o^C.

**
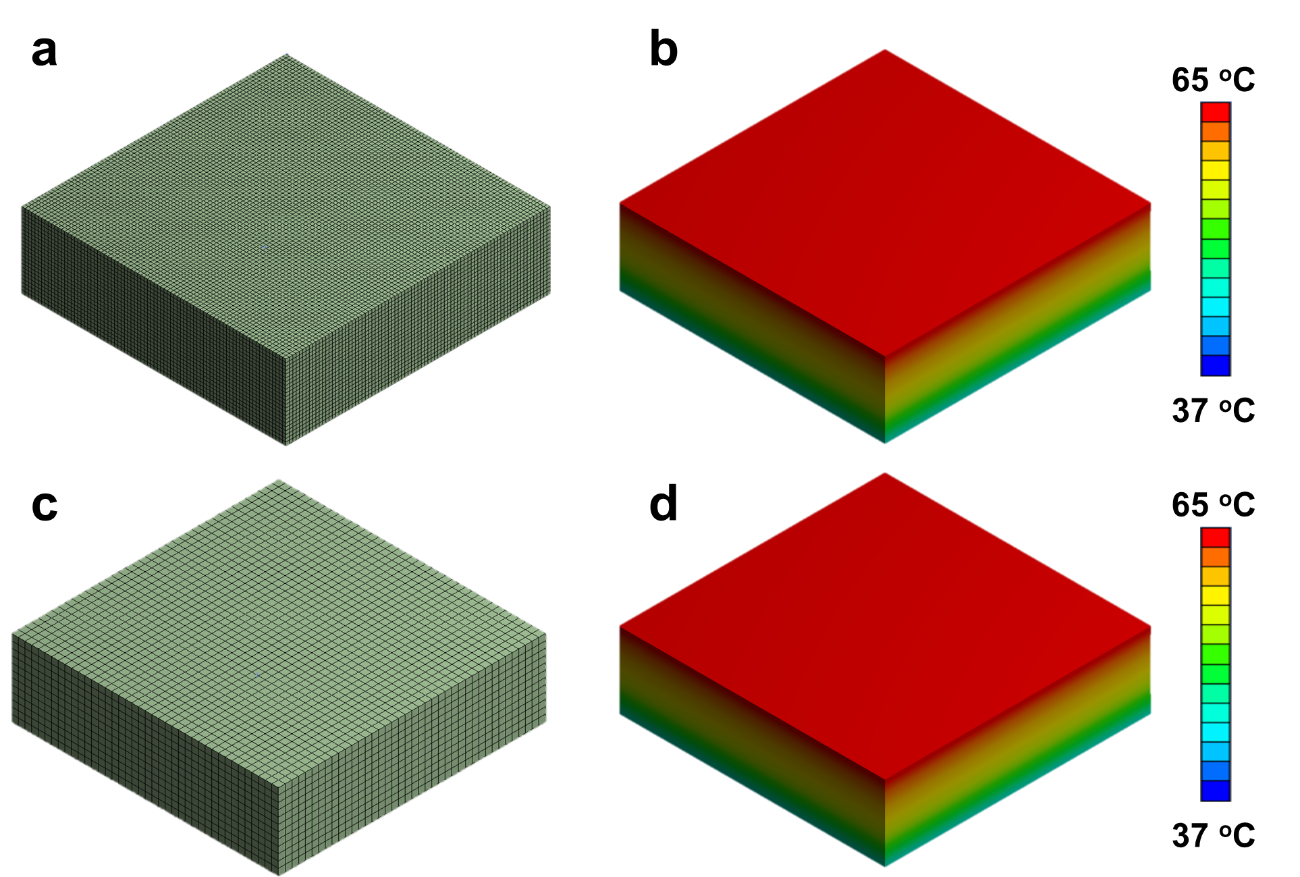
**

**Supplementary Fig. 5 | Independence test for grid in Supplementary Fig. 2-4. a,** Refined grid. **b,** The prediction of temperature distribution of **a**. **c,** Normal grid. **d,** The prediction of temperature distribution of **c**.

**
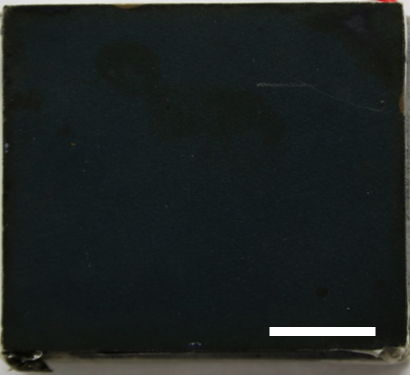
**

**Supplementary Fig. 6 | Photograph of a TE generator coated with a PT layer.** Scale bar: 1.0 cm.

**
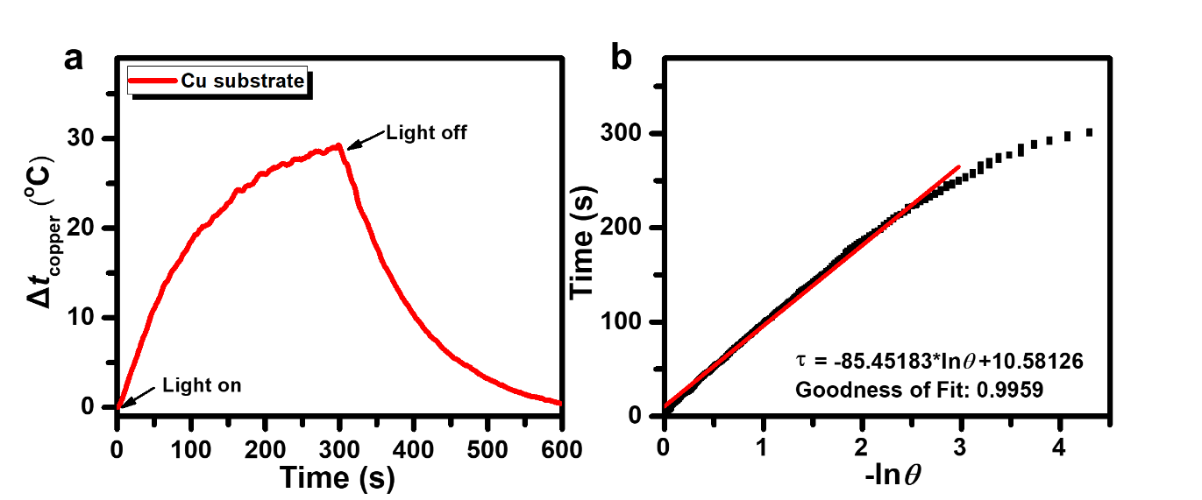
**

**Supplementary Fig. 7 | Calculation of photothermal conversion efficiency. a,** The curve of temperature change over time for the PT layer coating on a copper plate under on-off light irradiation. **b,** A linear fitting curve of time over ln*θ* obtained from the cooling curve of **a**. θ was defined as the ratio of Δ*t* to Δ*t*_max_.


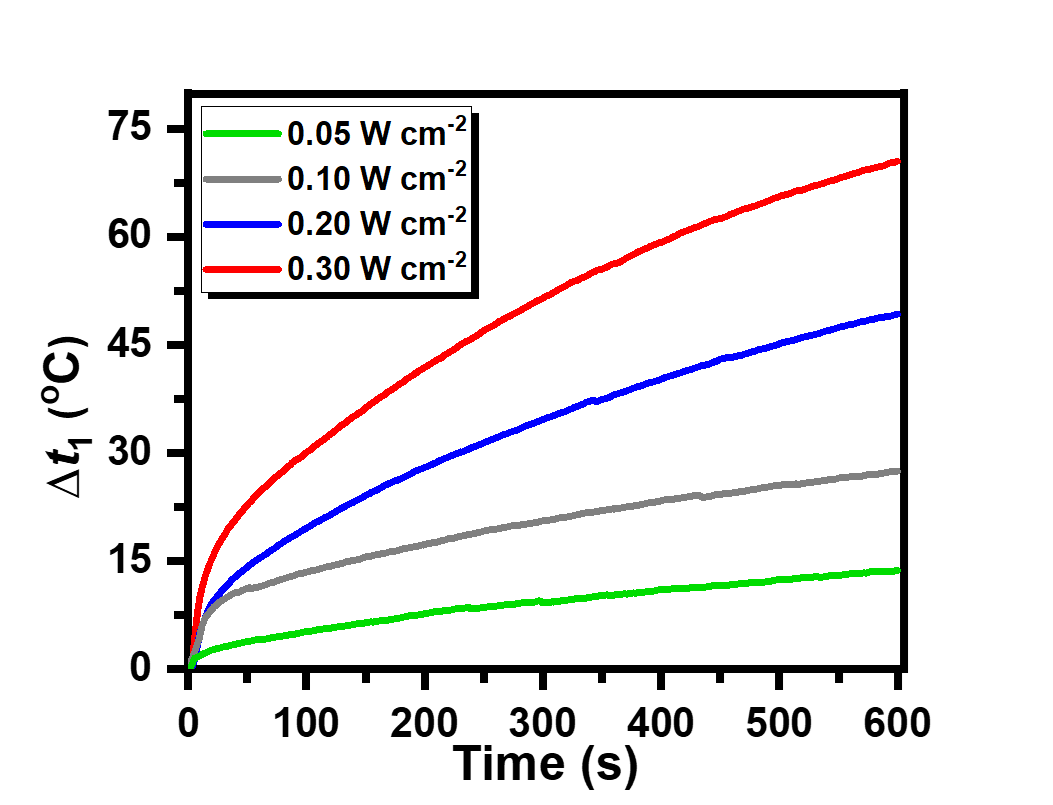


**Supplementary Fig. 8 | Temperature change of PT layer under the irradiation for 10 min.**


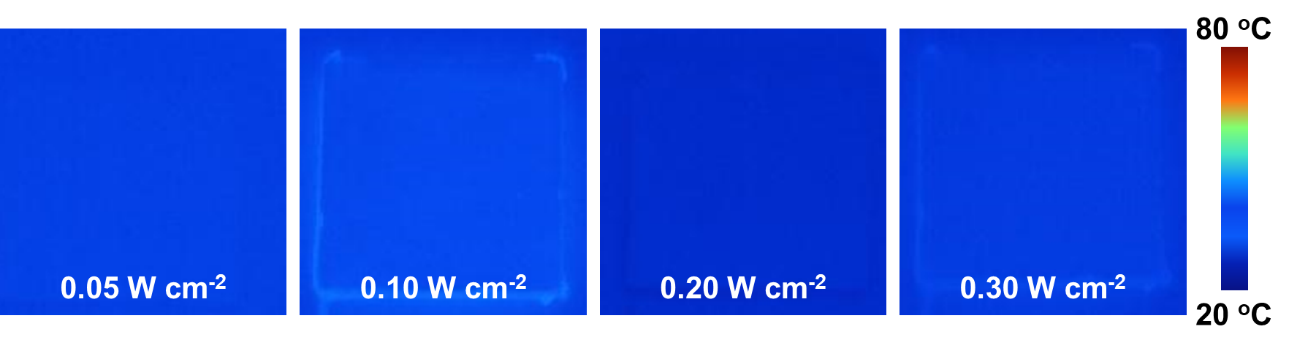


**Supplementary Fig. 9 | IR images of PT layer before NIR-II light irradiation.** These images were used as controls against the images in Fig. 2e. From left to right: The LPD of NIR-II light was 0.05 W cm^-2^, 0.10 W cm^-2^, 0.20 W cm^-2^, and 0.30 W cm^-2^, respectively.

**
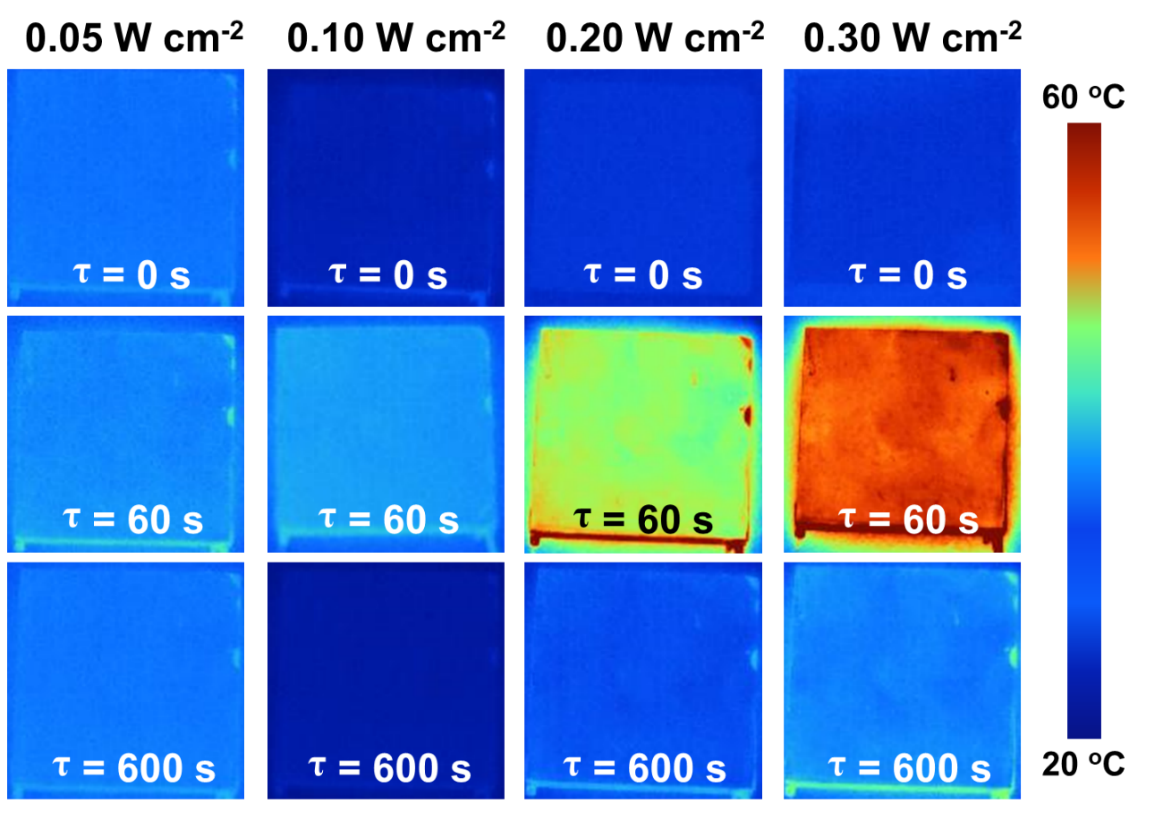
**

**Supplementary Fig. 10 | IR images of the TE generator coated with a PT layer.** From left to right: The LPDs of NIR-II light were 0.05 W cm^-2^, 0.10 W cm^-2^, 0.20 W cm^-2^, and 0.30 W cm^-2^, respectively. From top to bottom: At the beginning of light irradiation (τ=0 s), under light irradiation for 1 min (τ=60 s), and cooling for 9 min after turning off NIR-II light irradiation (τ=600 s).


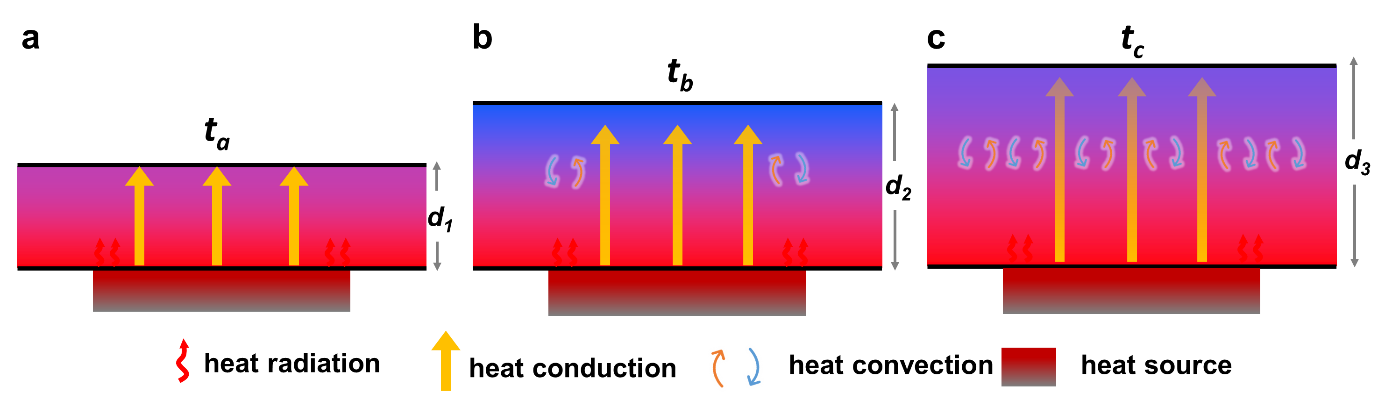


**Supplementary Fig. 11 | Scheme of the convective heat transfer with the gradual increase of *d*. a,** The heat transfer only induced by heat radiation and heat conduction in the case of small *d*_1_. **b,** The heat convection became non-negligible in heat transfer when *d*_2_ was increased to the critical value. **c,** The convection heat transfer played a determinant role in heat transfer and led to an increased heat transfer in total when *d*_3_ exceeded the critical value. *t*_2_(b)<*t*_3_(c)<*t*_1_(a).

**
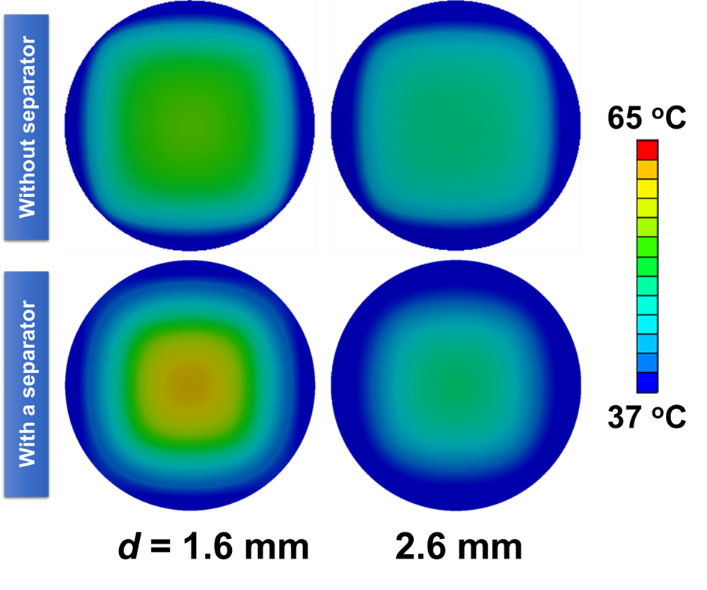
**

**Supplementary Fig. 12 | Predicated temperature distributions on the top surface of PTE converter with different *d* after NIR-II light irradiation for 10 min (corresponding to conduction region of Fig. 3c)**. Top: Without a quartz separator in the upper air layer. Bottom: With a quartz separator inserted in the upper air layer.


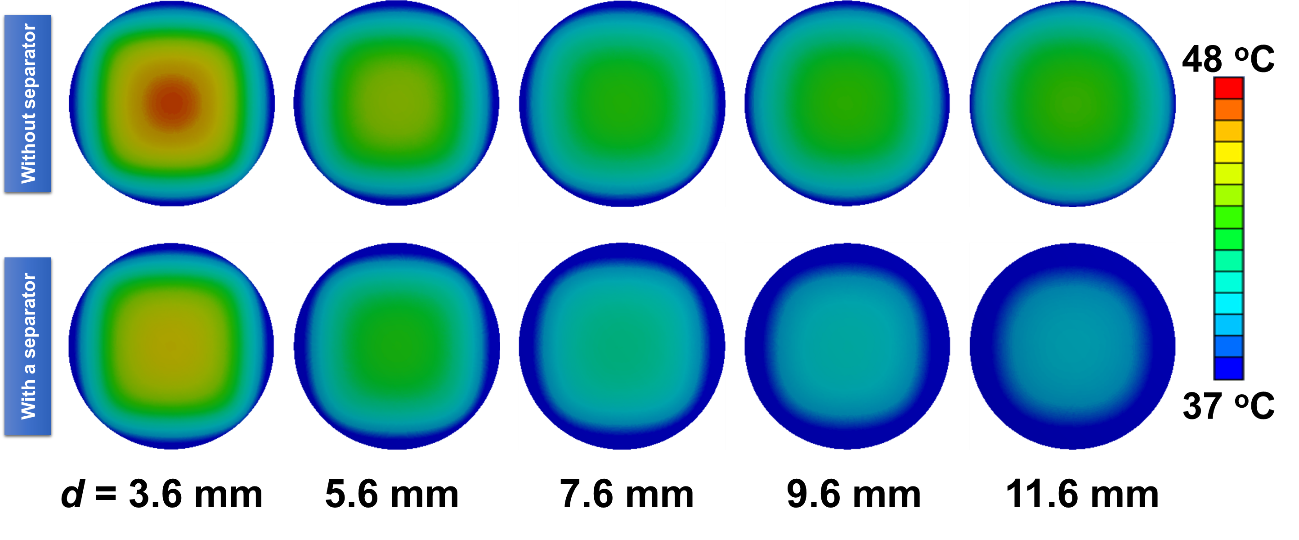


**Supplementary Fig. 13 | Prediction of temperature distributions on the top surface of PTE converter with different *d* after NIR-II ling irradiation for 10 min (corresponding to convection region of Fig. 3c-d)**. The heat convection could be restrained by the separator. Top row and Bottom row are converter with the upper air layer without a quartz separator or with a quartz separator.


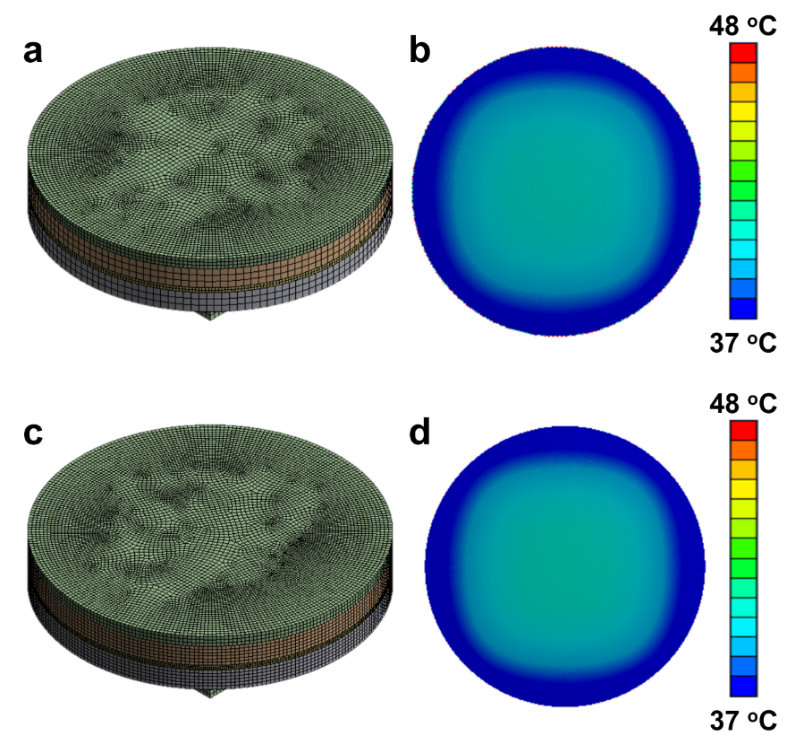


**Supplementary Fig. 14 | Independence test for grid in Supplementary Fig. S12-13. a,** Refined grid. **b,** The prediction of temperature distribution of **a**. **c,** Normal grid. **d,** The prediction of temperature distribution of **c**.


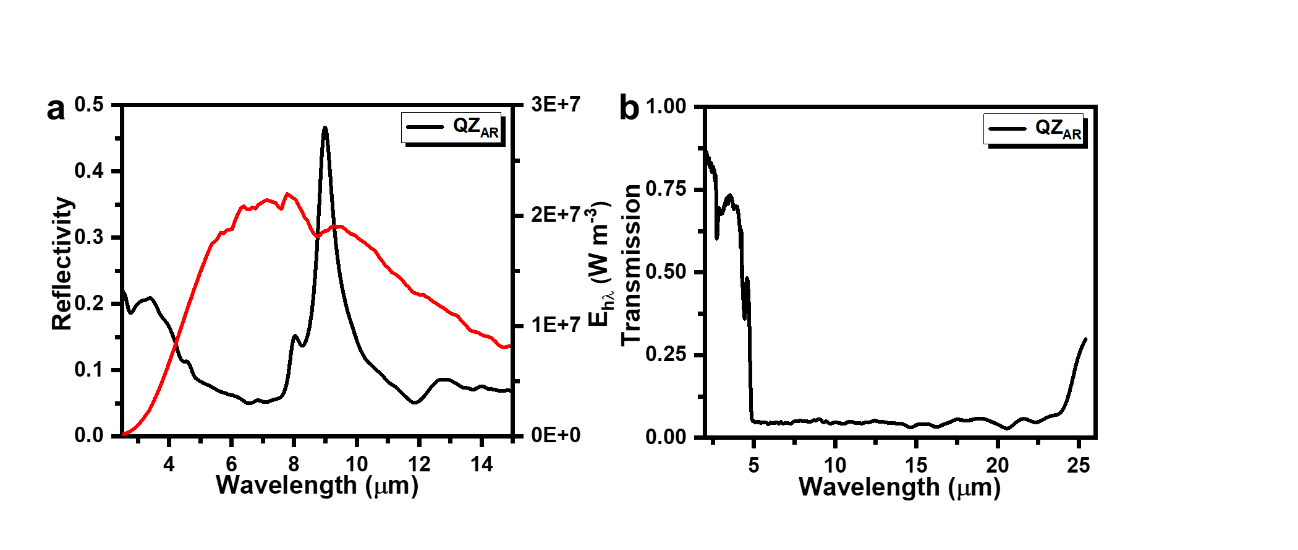


**Supplementary Fig. 15 | Reflectivity and transmission of quartz separator in the mid-infrared window. a.** Reflectivity of quartz separator (black line), and spectral radiant power of PT layer (E_hλ_, red line). **b.** Transmission of quartz separator. The temperature at PT layer was 105 ^o^C (corresponding to a LPD of 0.3 W m^-2^).

**
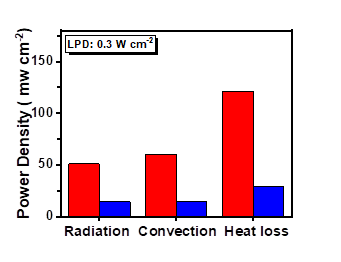
**

**Supplementary Fig. 16 | Estimation of radiation heat loss, convection heat loss, and total heat loss of unmodified PTE converter (red bar), and the PTE converter modified with an optimized upper layer (blue bar).** Total heat loss could be reduced by 70% in the case of upper layer modification.


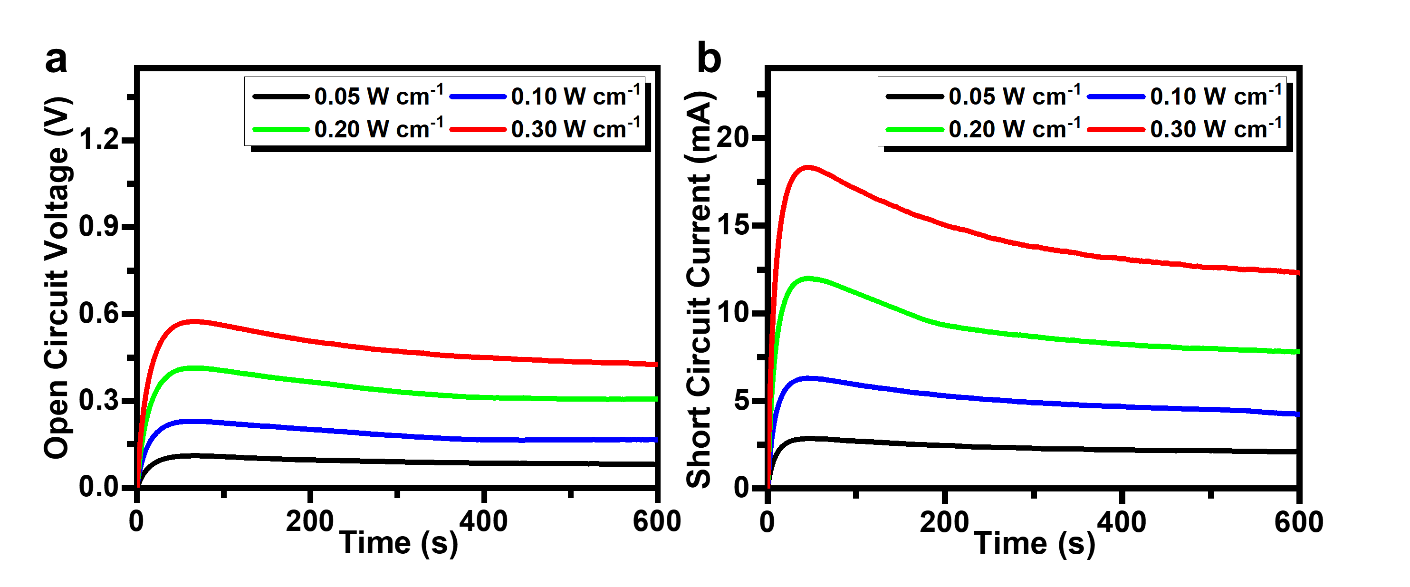


**Supplementary Fig. 17 | Open circuit voltage and short circuit current generated by PTE converter without upper layer.** **a,** Open circuit voltage over time under NIR-II light with different LPDs. **b,** Short circuit current over time under NIR-II light with different LPDs. They both increased firstly and then decreased and flattened over time, which was agreed with the predicted results of FEA (Supplementary Fig. S3) and non-steady-state heat transfer model (Supplemental discussion).


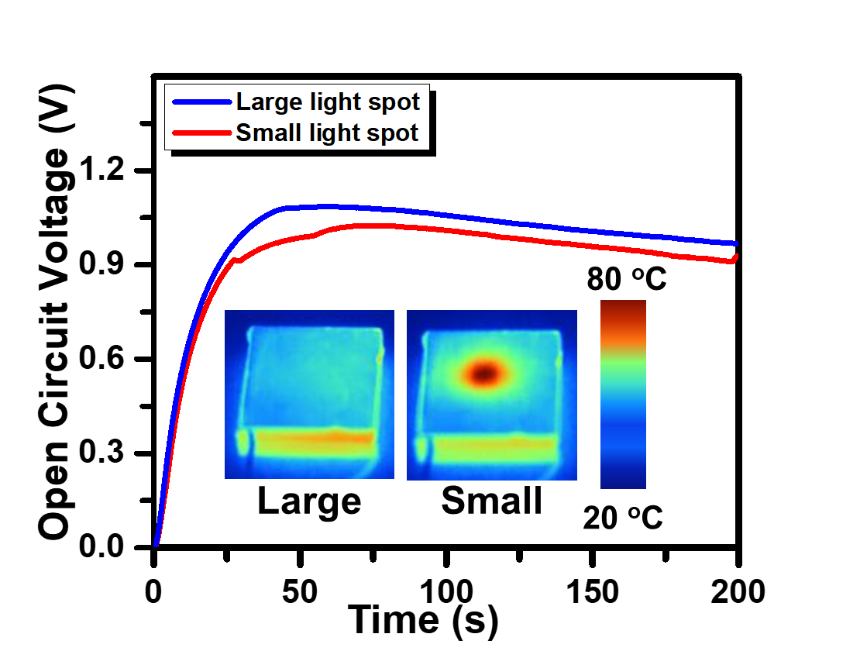


**Supplementary Fig. 18 | Influence of the light irradiation area.** The open circuit voltage over time of the same PTE converter under NIR-II light irradiation with larger light spot (blue) or smaller light spot (red) at a given LPD. It was suggested that more uniform light energy distribution was beneficial to higher energy conversion. Inset: The IR images of the TE generator coated with PT layer under NIR-II light irradiation with different illumination area. The LPD of irradiation was 0.3 W cm^-2^.


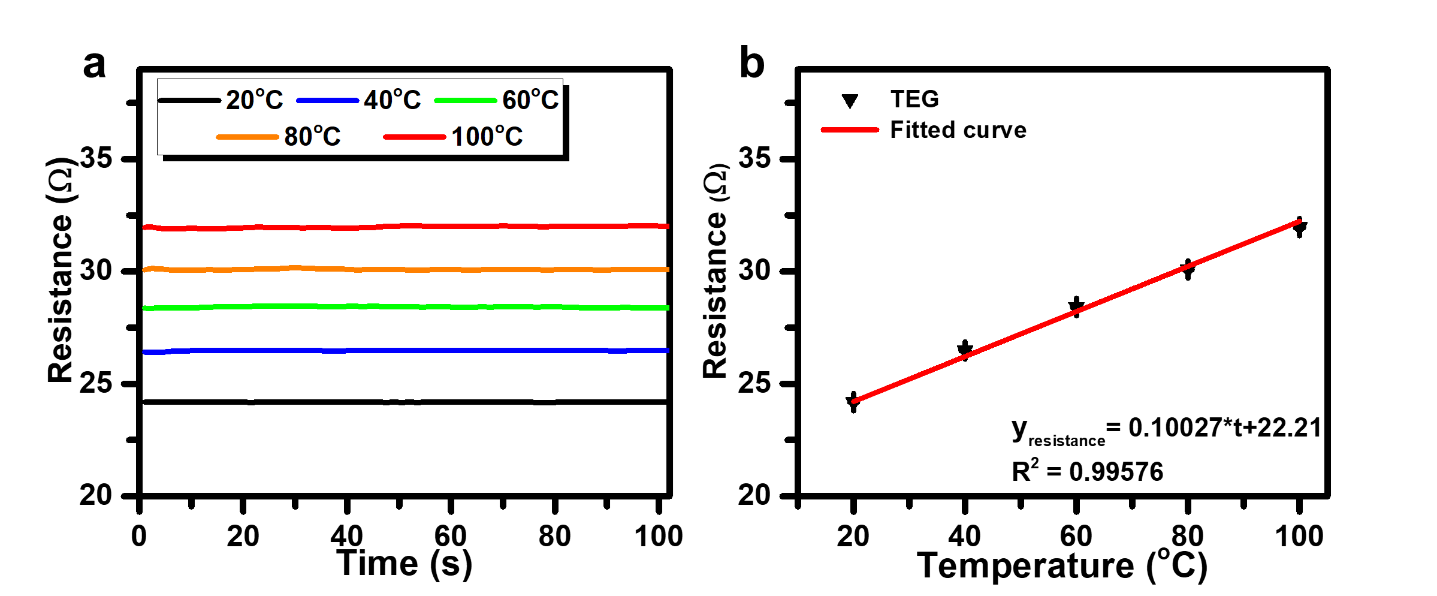


**Supplementary Fig. 19 | Electrical performance of TE generator. a,** The internal resistance of TE generator at different temperatures. **b,** The fitted curve of resistance as function of temperature. The resistance at different temperature are 24.190±0.006 Ω (20^o^C), 26.481±0.019 Ω(40^o^C), 28.421±0.027 Ω(60^o^C), 30.091±0.024 Ω(80^o^C), and 31.979±0.042 Ω(100^o^C) . (n=6).


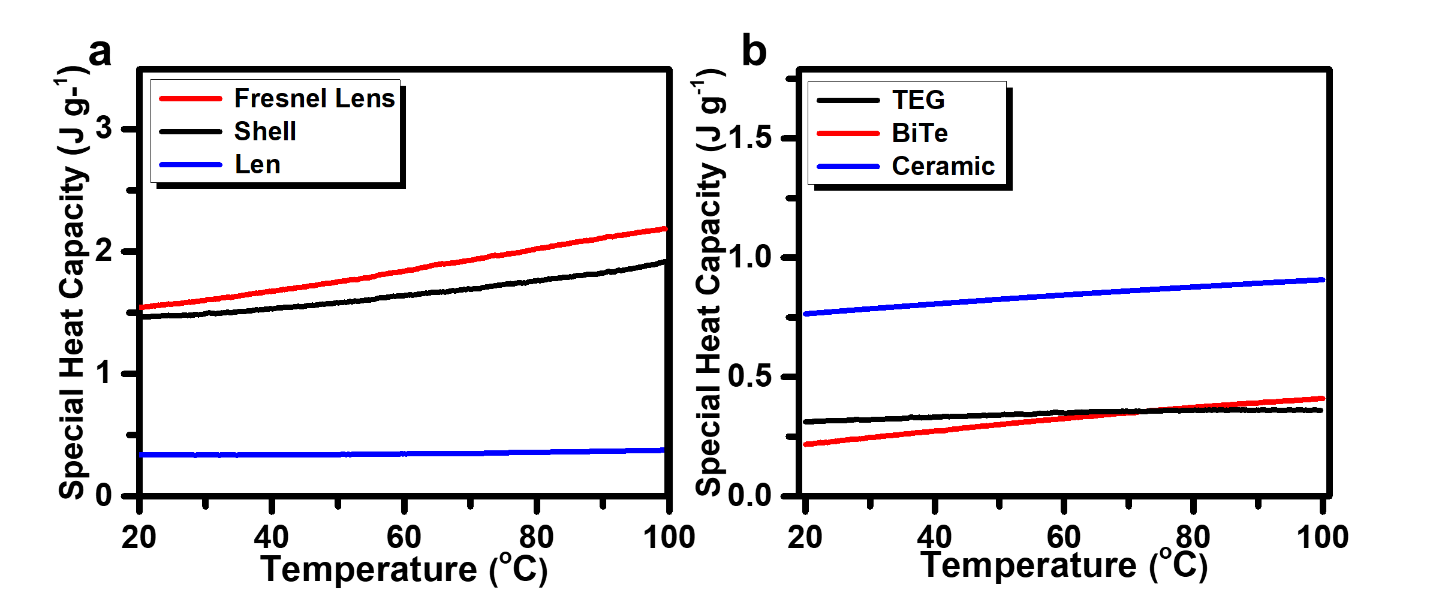


**Supplementary Fig. 20 | Thermal parameters of Bio-PS. a,** The specific heat capacities of the Fresnel lens, the 3D-printed shell and fin. **b,** The specific heat capacity of the TE generator.

**
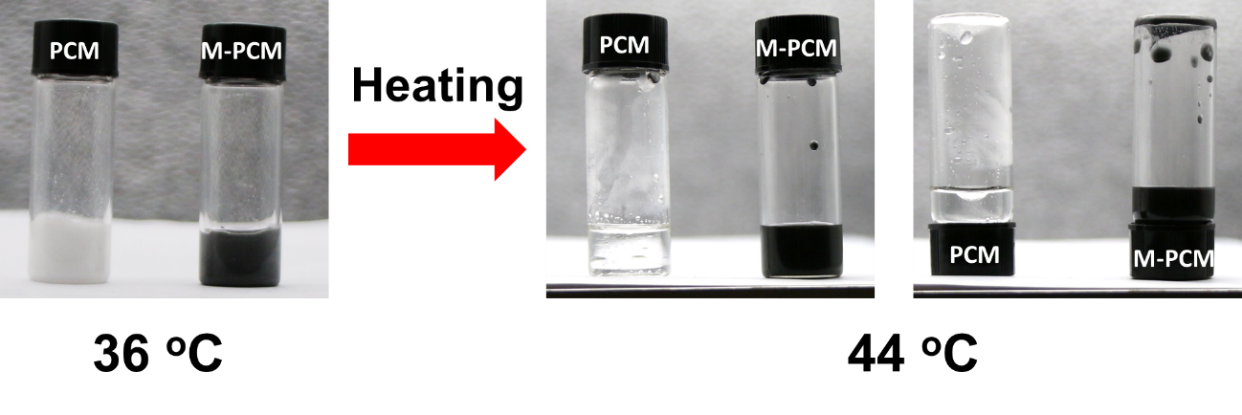
**

**Supplementary Fig. 21 | Photographs of PCM and M-PCM stored in glass vessels.** Both of them were melted and became fluidic under heating from 36 ^o^C to 44^o^C.


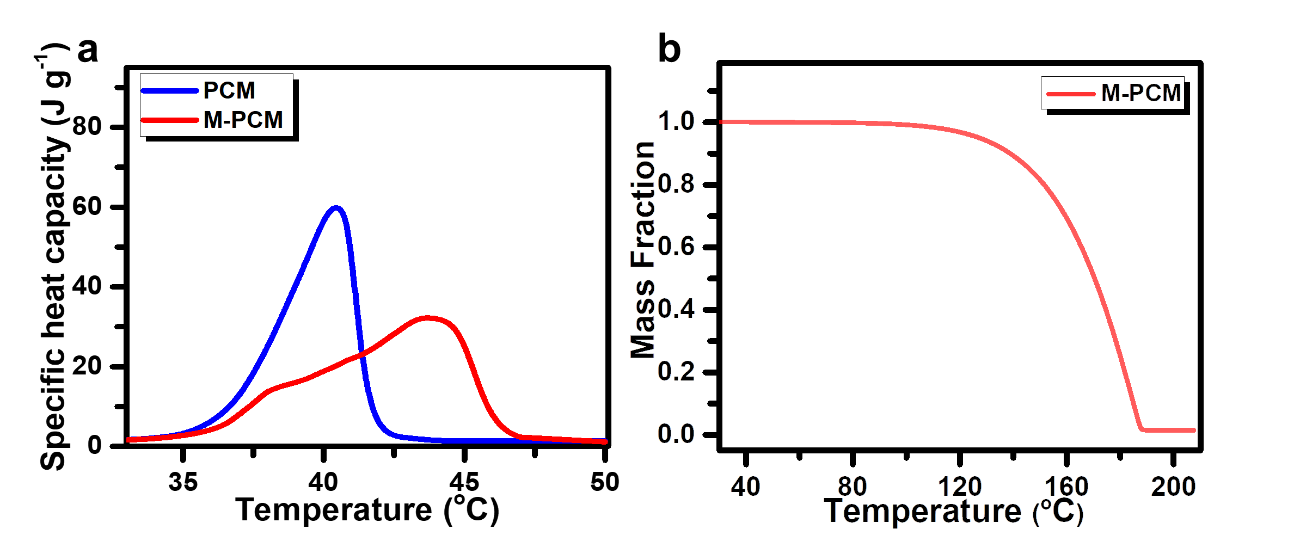


**Supplementary Fig. 22 | Thermal properties of PCM and M-PCM. a,** The specific heat capacity of the PCM and the M-PCM over temperature. **b,** Thermogravimetric analysis (TGA) of the M-PCM, which illustrated M-PCM within the temperature range in this project was thermally stable.


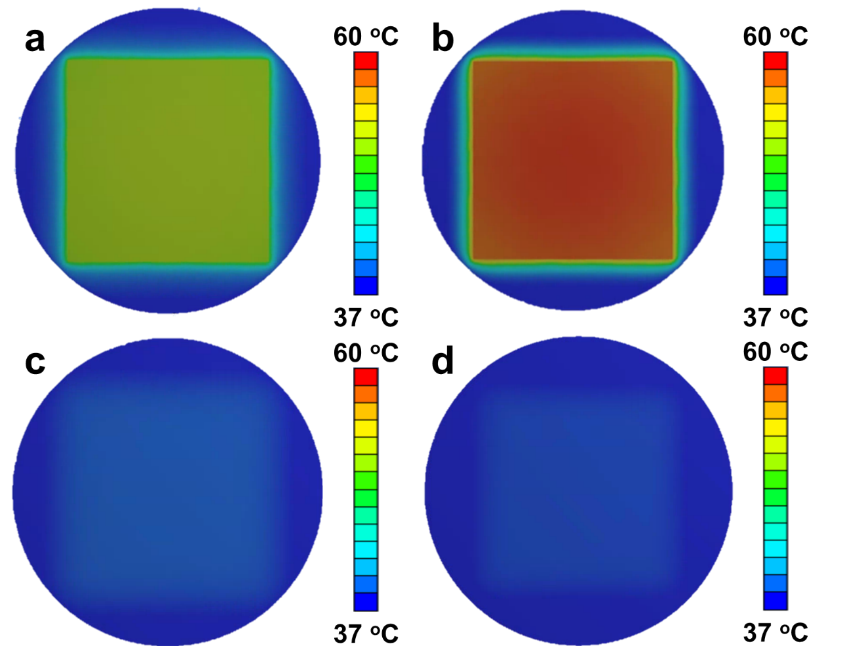


**Supplementary Fig. 23 | Simulated temperature distribution in the lateral direction of the bottom layer under heat flow for 10 min. a,** The top surface of the bottom layer with PCM. **b,** The top surface of the bottom layer with M-PCM. **c,** The bottom surface of the bottom layer with PCM. **d,** The bottom surface of the bottom layer with M-PCM.


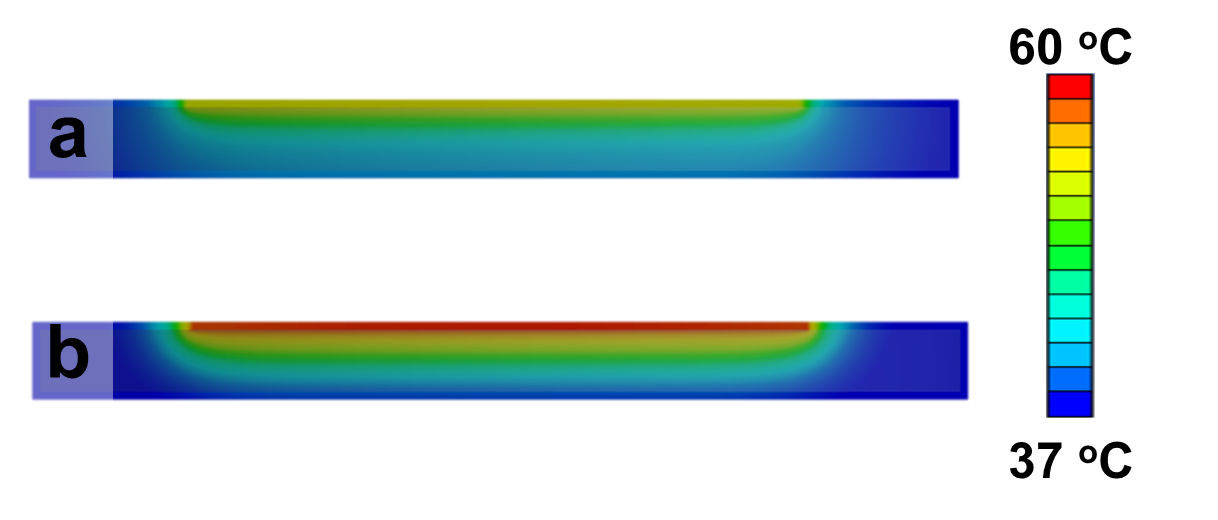


**Supplementary Fig. 24 | Simulated temperature distribution in the vertical direction of the bottom layer under heat flow for 10 min. a,** The bottom layer with PCM. **b,** The bottom layer with M-PCM.

**
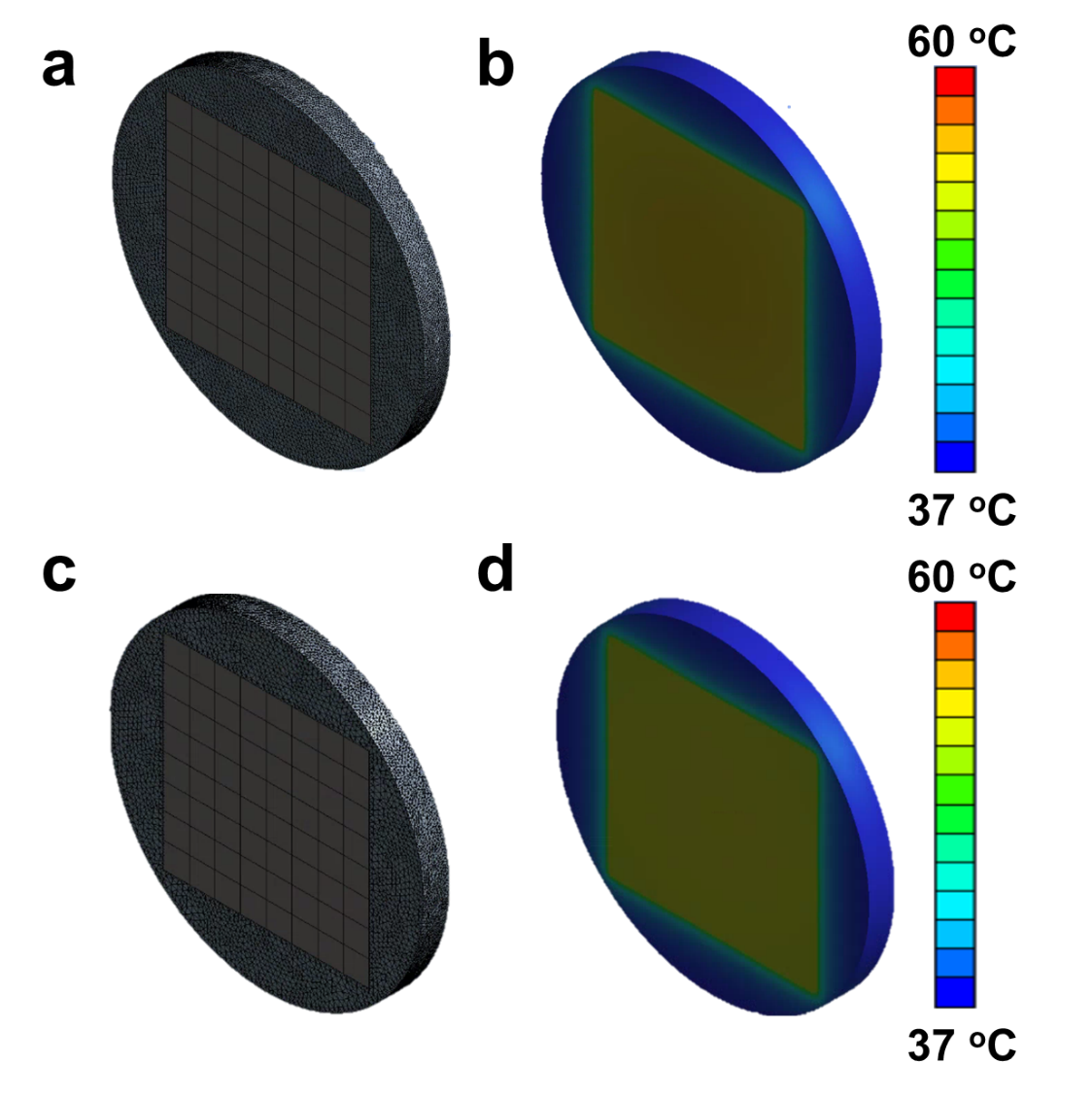
**

**Supplementary Fig. 25 | Independence test for grid in Supplementary Fig. 4d and Fig. 23-24. a,** Refined grid. **b,** The prediction of temperature distribution of **a**. **c,** Normal grid. **d,** The prediction of temperature distribution of **c**.


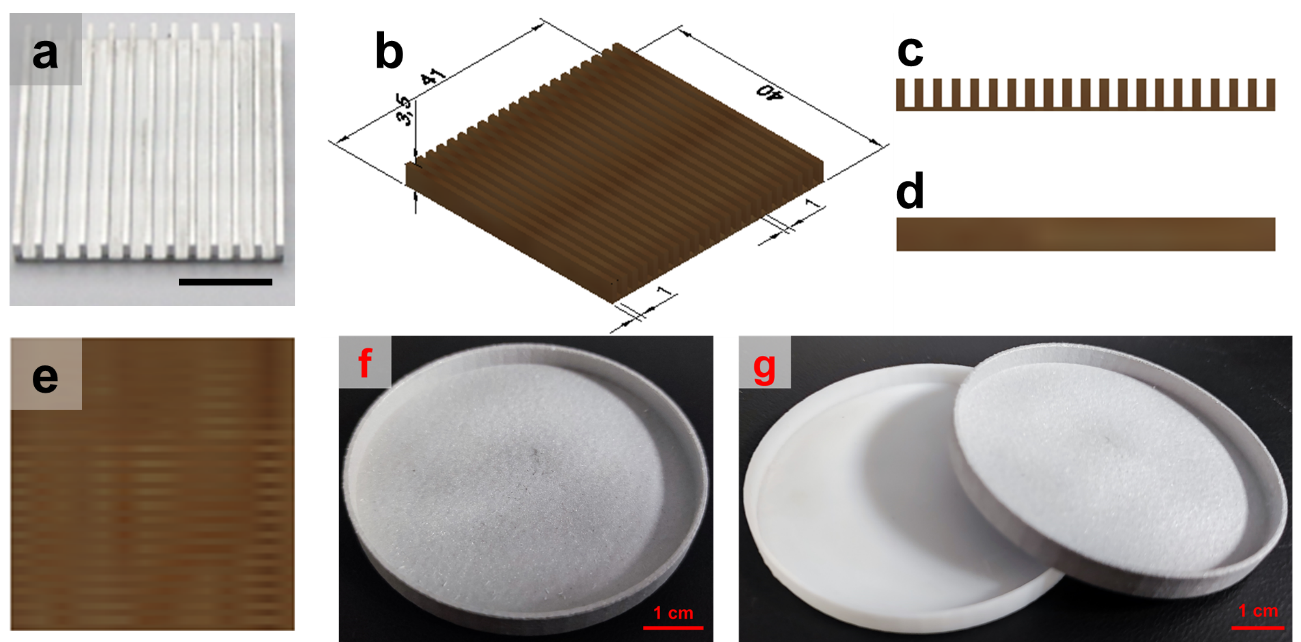


**Supplementary Fig. 26 | Characterization of the cooling fin. a,** The image of the fin. Scale bar: 1.0 cm. **b,** The design and parameters of the fin. **c,** The side view of the fin. **d,** The front view of the fin. **e,** The top view of the fin. **f-g,** Image of the aluminum alloy base (f) and resin base (g).


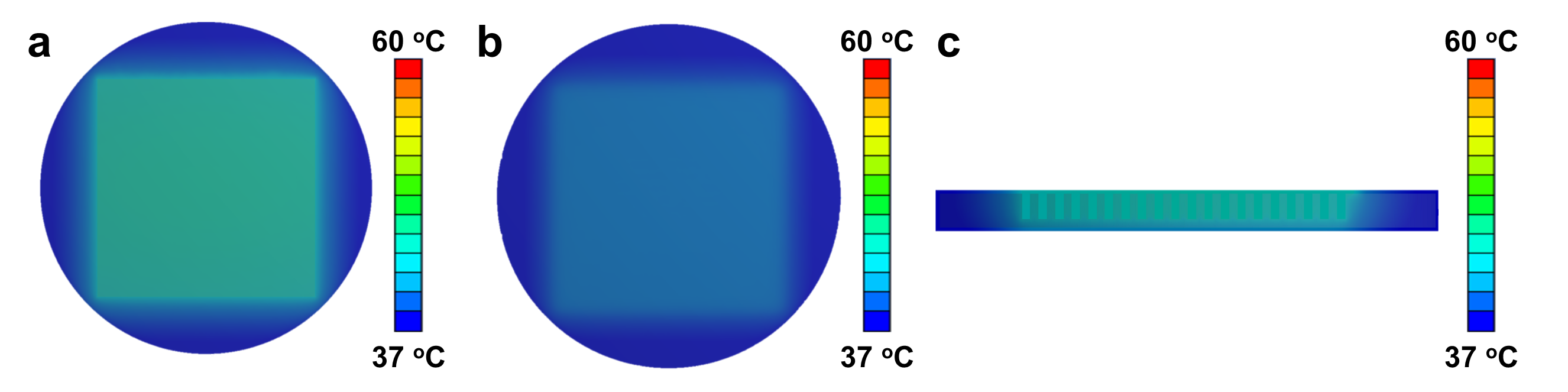


**Supplementary Fig. 27 | Simulated temperature distribution of the bottom layer which was put on a cooling fin under heat flow for 10 min. a,** Top surface, **b,** Bottom surface, and **c,** The section of the bottom layer.

**
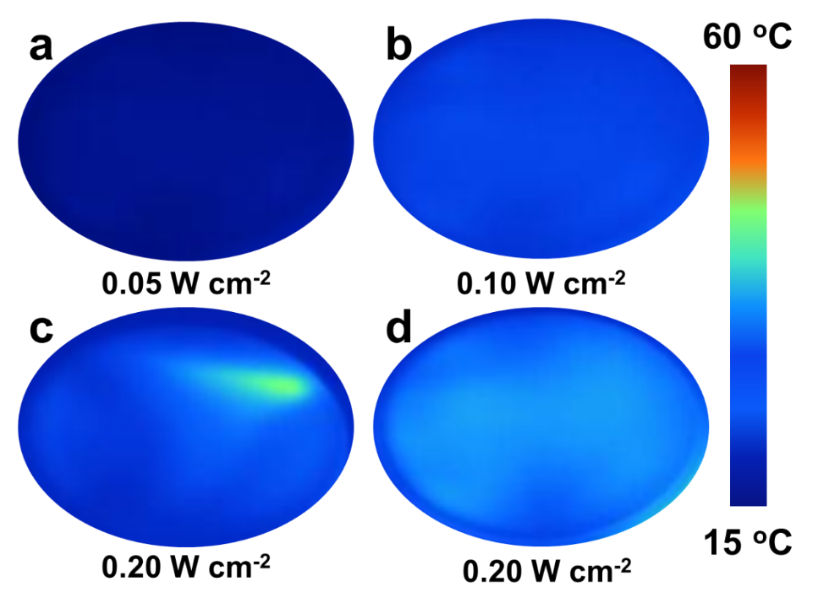
**

**Supplementary Fig. 28 | IR images of the Bio-PS’ top surface under NIR-II light with different LPDs. a-d,** The maximum Δ*t*_0_ referring to the temperature change before and after NIR-II light irradiation was observed as 1^o^C, 1.5^o^C, 2.4^o^C, and 4.3^o^C under light irradiation with LPD of 0.05W cm^-2^ (**a**), 0.10 W cm^-2^ (**b**), 0.20 W cm^-2^ (**c**), 0.30 W cm^-2^ (**d**) for 10 min.

**
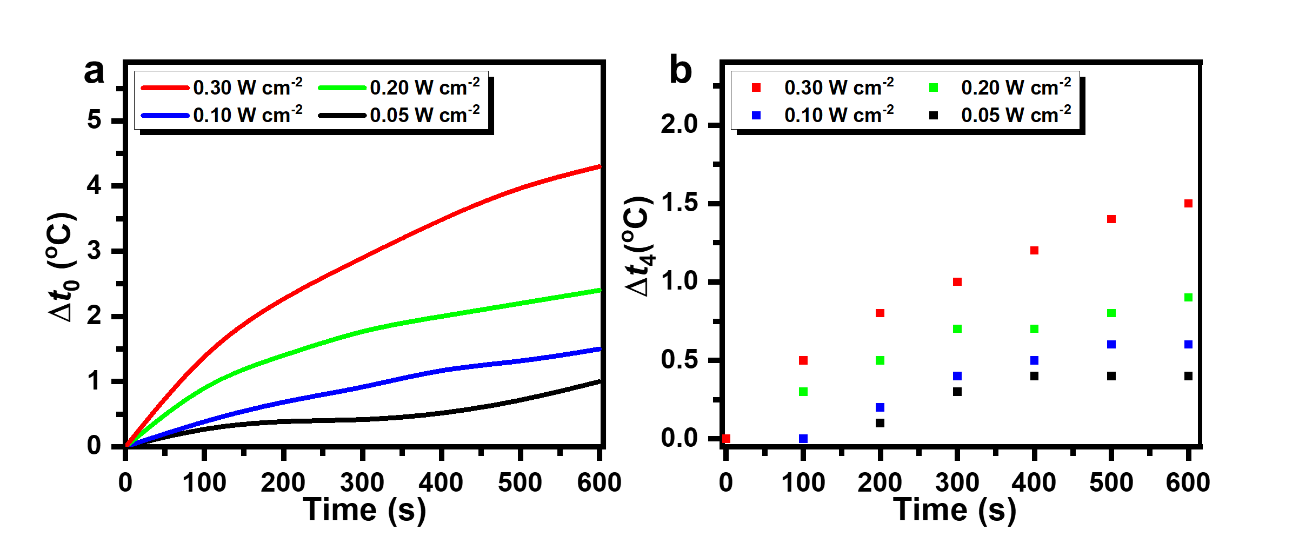
**

**Supplementary Fig.29 | Evaluation of the temperature safety of Bio-PS during 10-min light irradiation. a,** The real-time Δ*t*_0_ over time. **b,** The curve of Δ*t*_4_ over time. Both Δ*t*_0_ and Δ*t*_4_ were less than 5^o^C in this process.

**
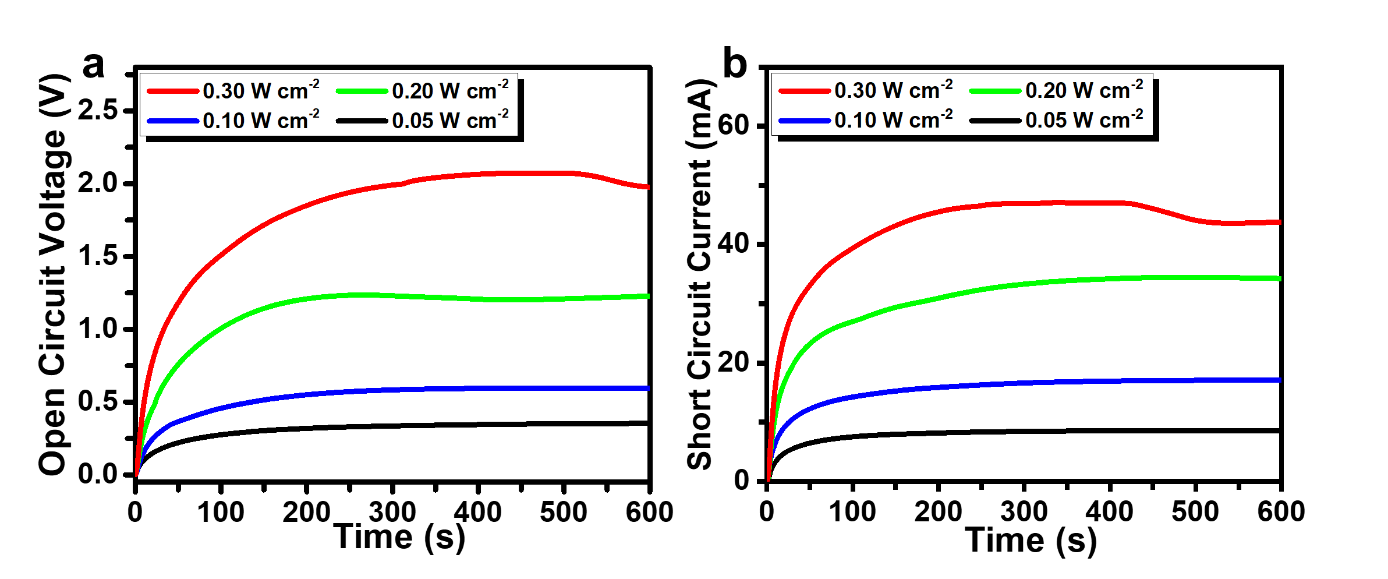
**

**Supplementary Fig.30 | Output energy of the Bio-PS during 10-min light irradiation. a,** Open circuit voltage of Bio-PS under NIR-II light irradiation with different LPDs. **b,** Short circuit current of Bio-PS under NIR-II light irradiation with different LPDs.

**
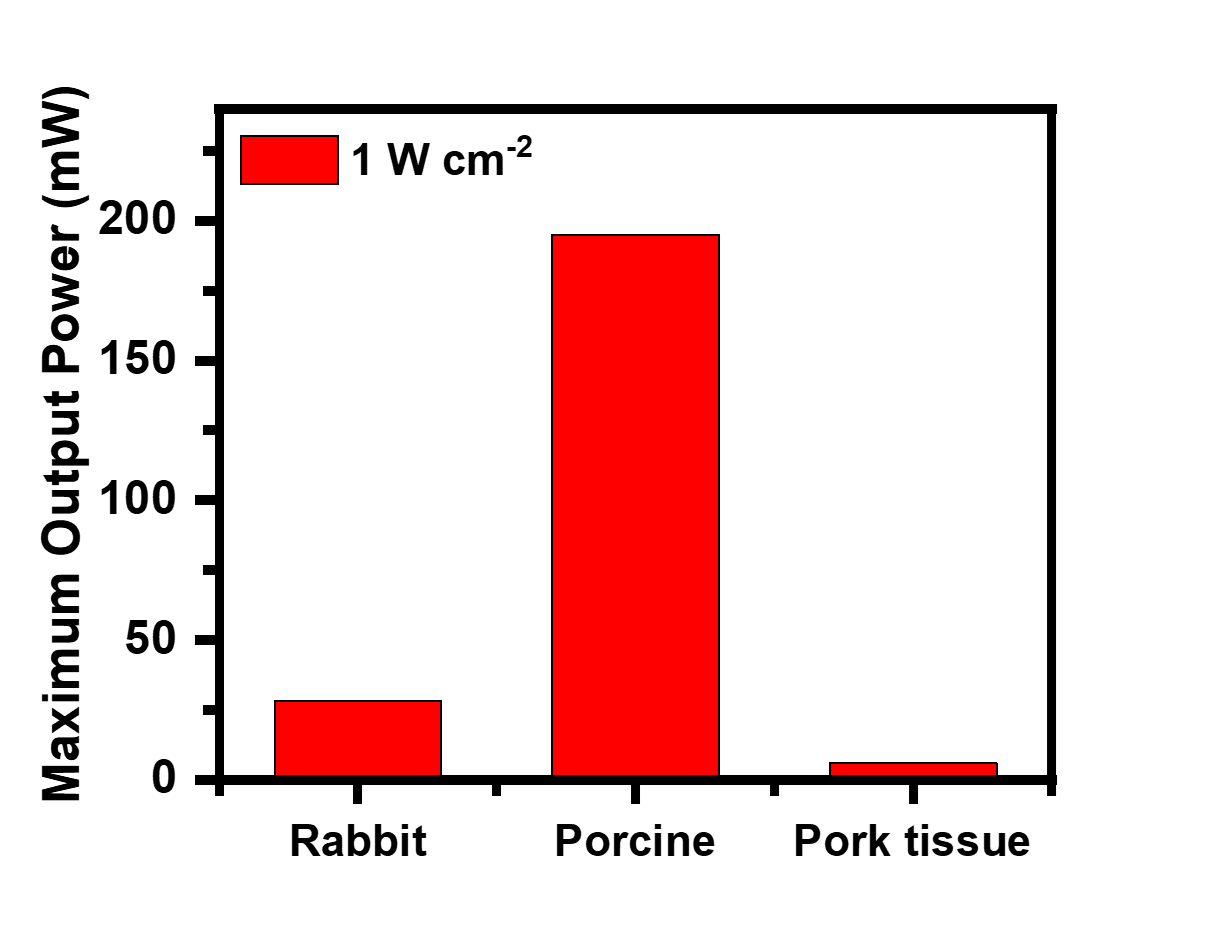
**

**Supplementary Fig.31 | Maximum output power of Bio-PS covered by rabbit tissue with thickness of 8.5 mm, porcine with thickness of 3.5 mm, and pork tissue with thickness of 20 mm.**

**
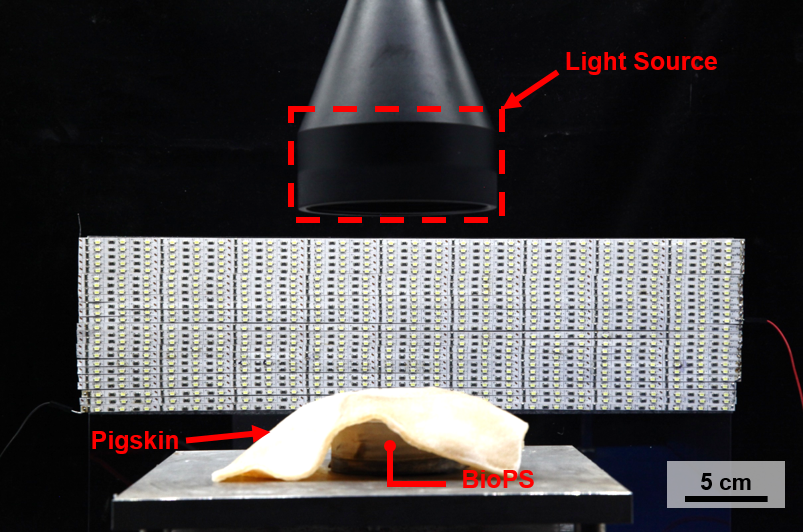
**

**Supplementary Fig. 32 |** **Photograph of a (red line) covered by piece of 3.5-mm-thickness pigskin (red arrow), which was connected with an array of 648 LEDs (corresponding to Fig 5b) and then subjected to the light source (red dotted box).**


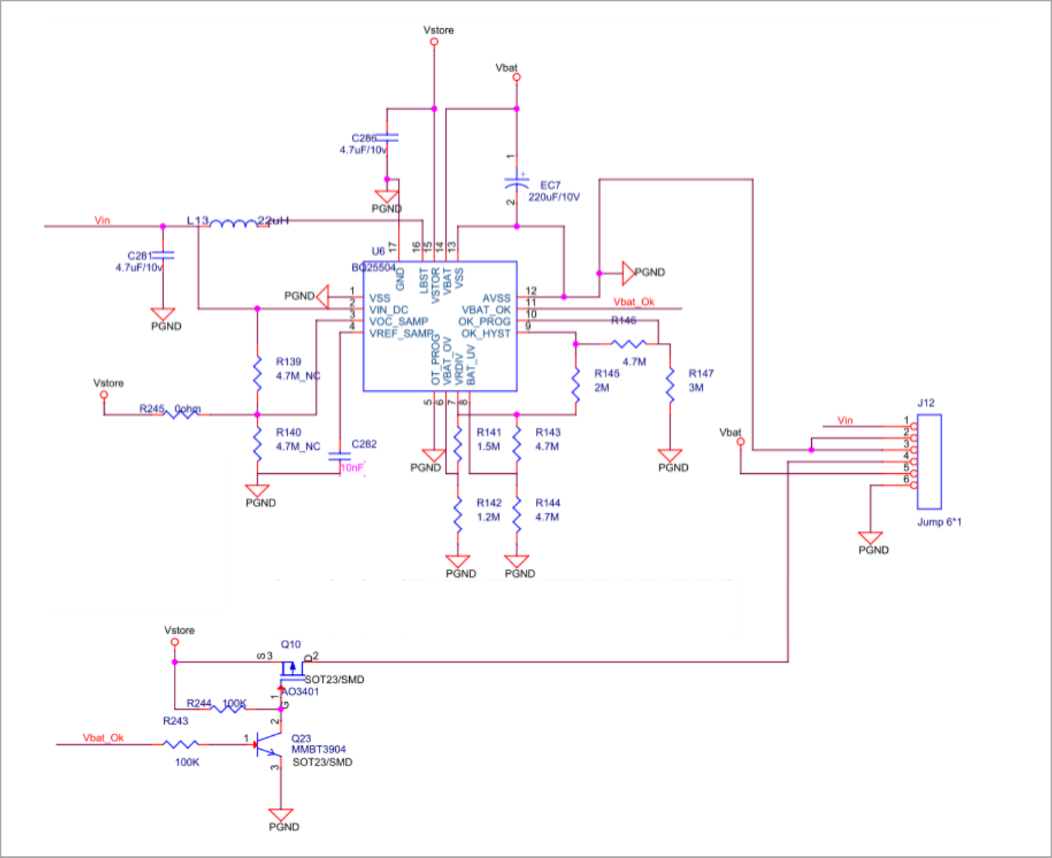


**Supplementary Fig. 33 | Circuit diagram of the step-up transformer.**

**
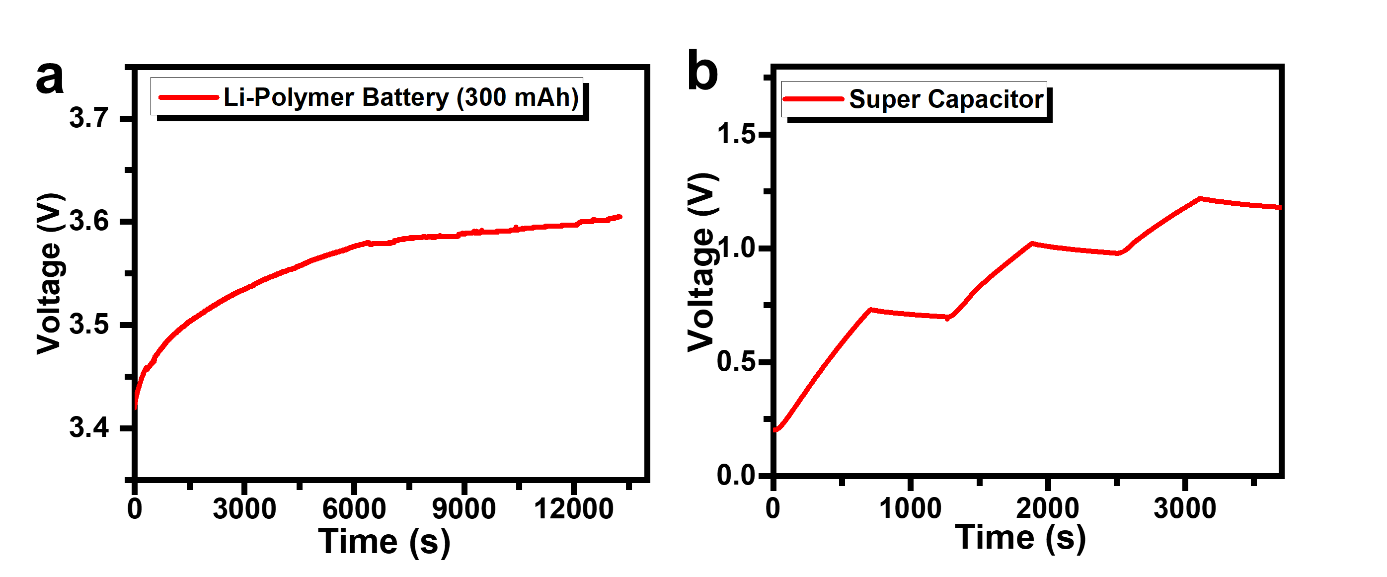
**

**Supplementary Fig. 34 | Direct power supply for charging** **polymer lithium battery (a), and** **super-capacitor (b) by Bio-PS.** Bio-PS was covered by a piece of 3.5-mm-thickness pigskin. The capacities of battery and super-capacitor were 300 mAh and 20 F, respectively. The LPD of NIR-II light was 1.0 W m^-2^. Both light irradiation time and interval time were 10 minutes.

**
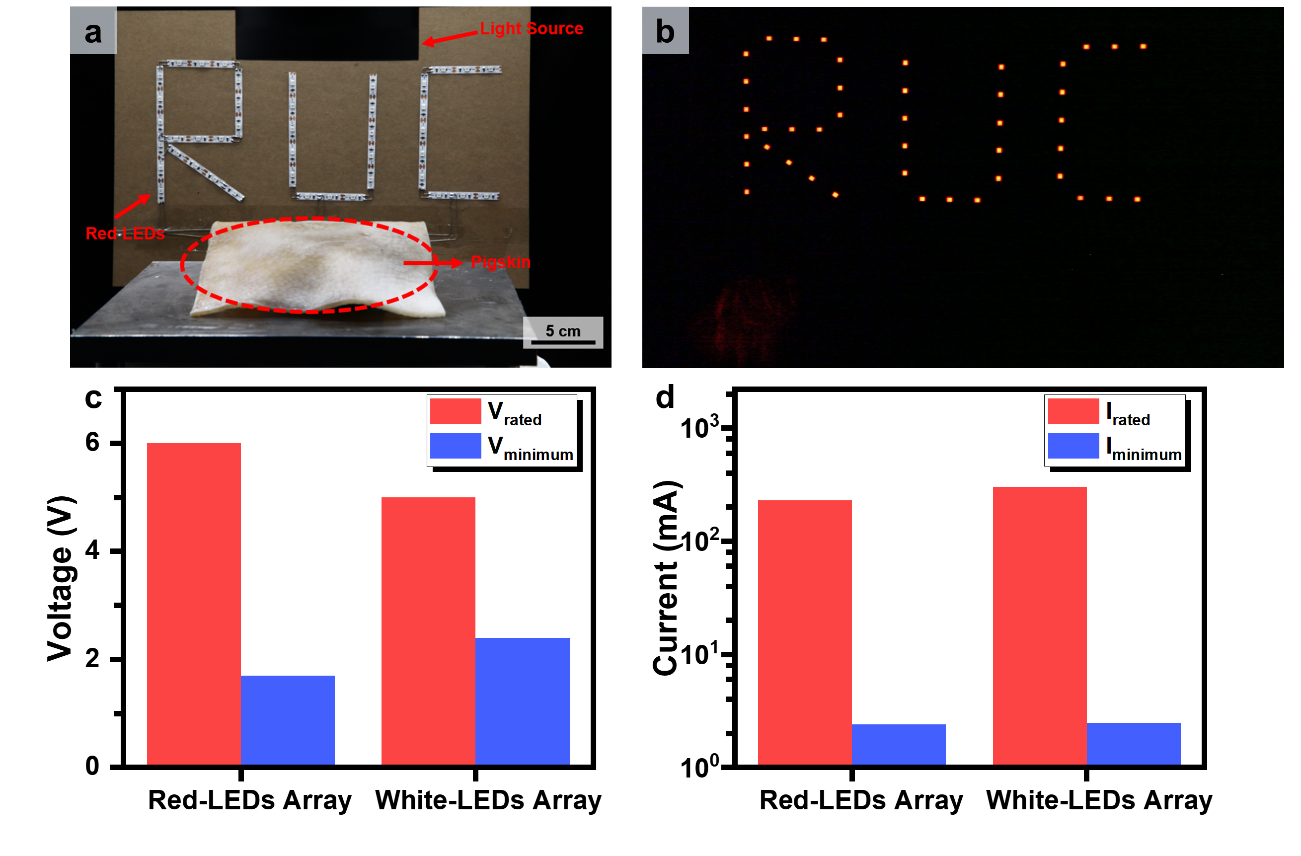
**

**Supplementary Fig. 35 | Photographs of direct power supply for** **high-power red LEDs patterned into a “RUC” character by Bio-PS. a,** The photograph of a Bio-PS covered by a piece of 3.5-mm-thickness pigskin (red dotted box), the light source, and a high-power red LEDs pattern as a “RUC” character. **b,** The photograph of direct power supply for the RUC patterned LEDs by Bio-PS. The LPD of NIR-II light was 1.0 W m^-2^. **c-d,** The electrical parameters of the white and red LEDs array in the Supplementary Fig. 32 and 35. The rated voltage and current of the red-LEDs array and white-LEDs array were 6 V, 5 V, and 230 mA, 300 mA, respectively. Meanwhile, the minimum voltage and current are 1.7 V, 2.4 V, and 2.4 mA, 2.47 mA, respectively. The numbers of red-LEDs array and white-LEDs array are 43 and 351.

**
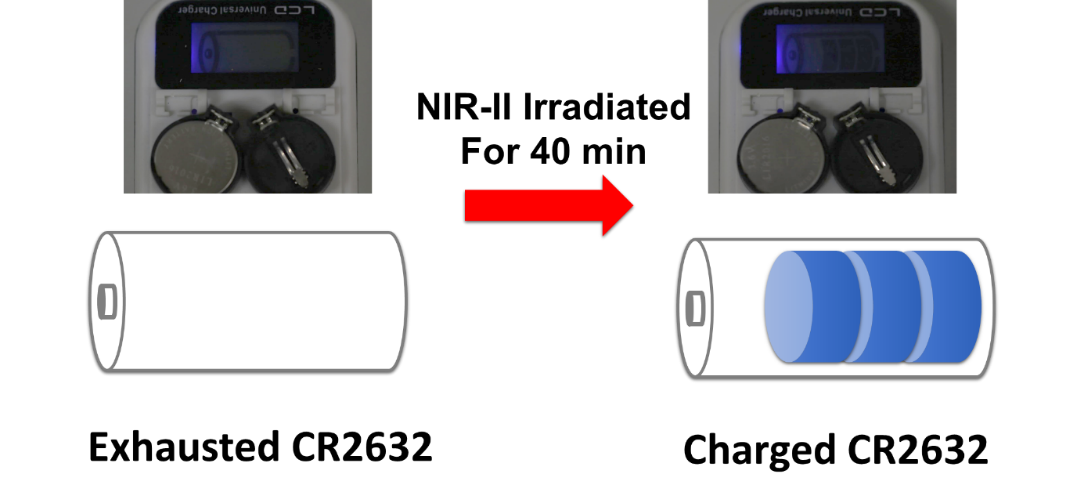
**

**Supplementary Fig. 36 | Direct power supply for charging** **button cell by Bio-PS.** Bio-PS received NIR-II light irradiation through a piece of 3.5-mm-thickness pigskin. The LPD of NIR-II light was 1.0 W m^-2^. The light irradiation was lasted for 10 min with an interval of 10 min.

**
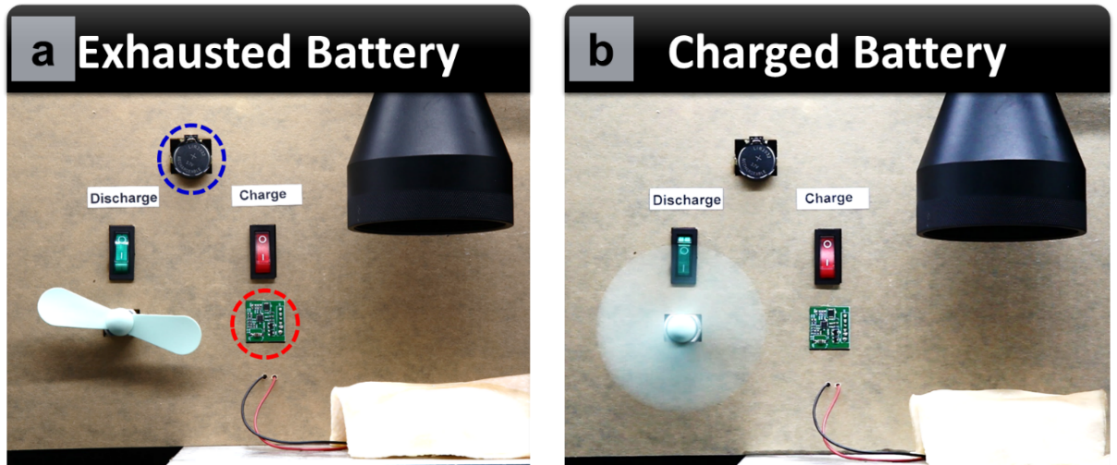
**

**Supplementary Fig. 37 | Indirect power supply for recharging battery by Bio-PS, and further driving fan. a,** The photograph of Bio-PS covered by a piece of 3-mm-thickness pigskin, a light source, a special step-up transformer (red circle), an exhausted battery (blue circle), two switches, and a high-power fan. **b,** After 40-min NIR-II light irradiation, the battery successfully drove the fan. The LPD of NIR-II light was 1.0 W m^-2^. The light irradiation was lasted for 10 min with an interval of 10 min.

**
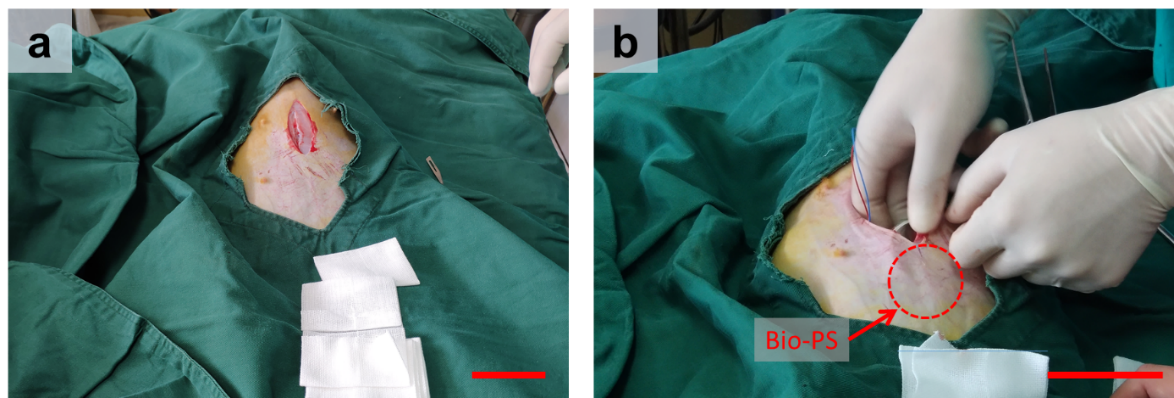
**

**Supplementary Fig. 38 | Implantation surgery.** **a,** The abdominal epithelial tissue of rabbit was cut with a scalpel**. b,** Bio-PS was placed in the rabbit’s abdominal cavity. The hair of the irradiation region had been removed. Scale bar: 5.0 cm.

**
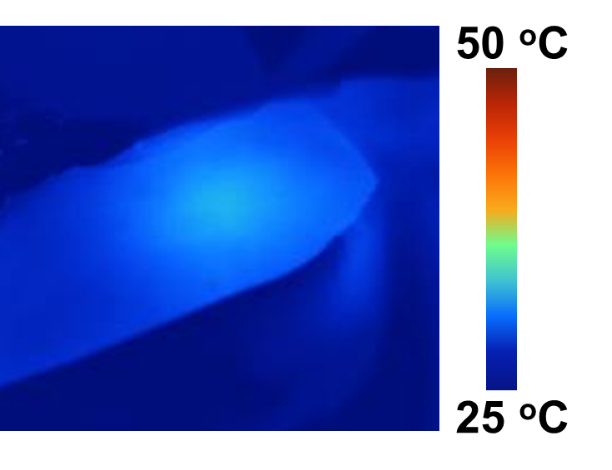
**

**Supplementary Fig. 39 | Temperature distribution of rabbit skin before NIR-II light irradiation (corresponding to Fig. 5d).**


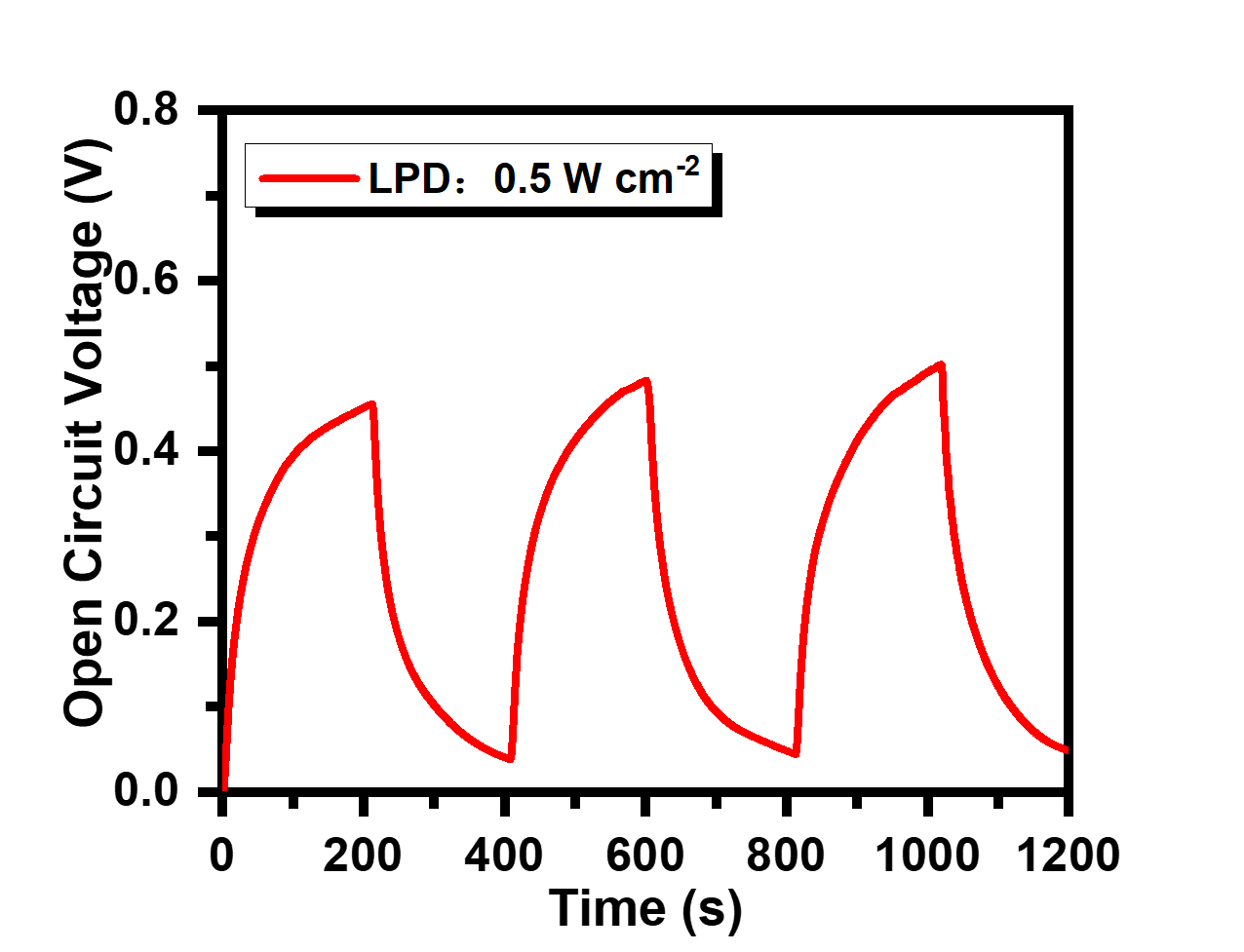


**Supplementary Fig. 40 | Open circuit voltage under on-off NIR-II light irradiation in vivo.** Bio-PS had been implanted in the rabbit’s abdominal cavity. The LPD of NIR-II light was 0.5 W m^-2^.


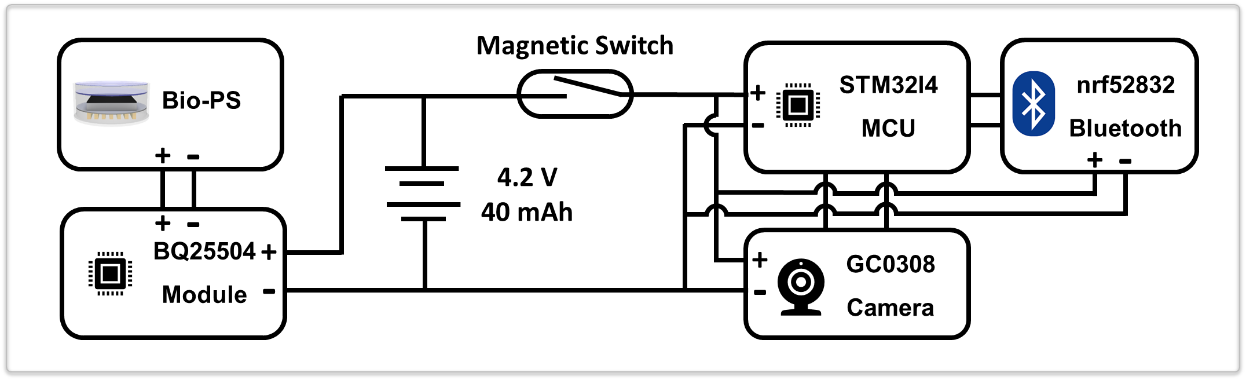


**Supplementary Fig. 41 | Circuit diagram of the wireless camera implanted in rabbit’s body.**


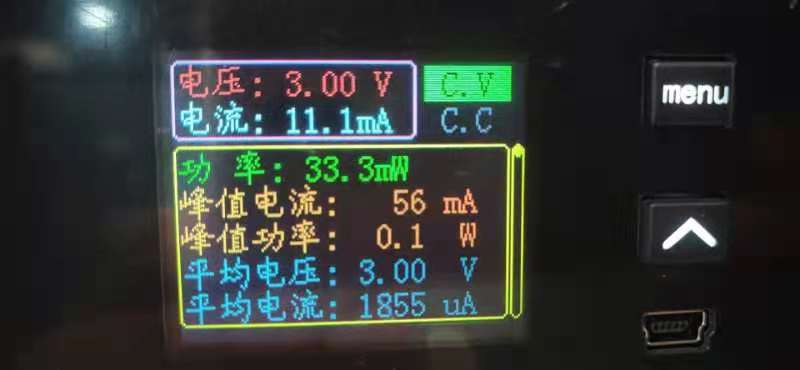


**Supplementary Fig. 42** | **Image of the power consumption of the implanted wireless camera.** It was revealed that the maximum consumption of such a wireless camera was over 100 mW with an average energy consumption of 55.65 mW.

**
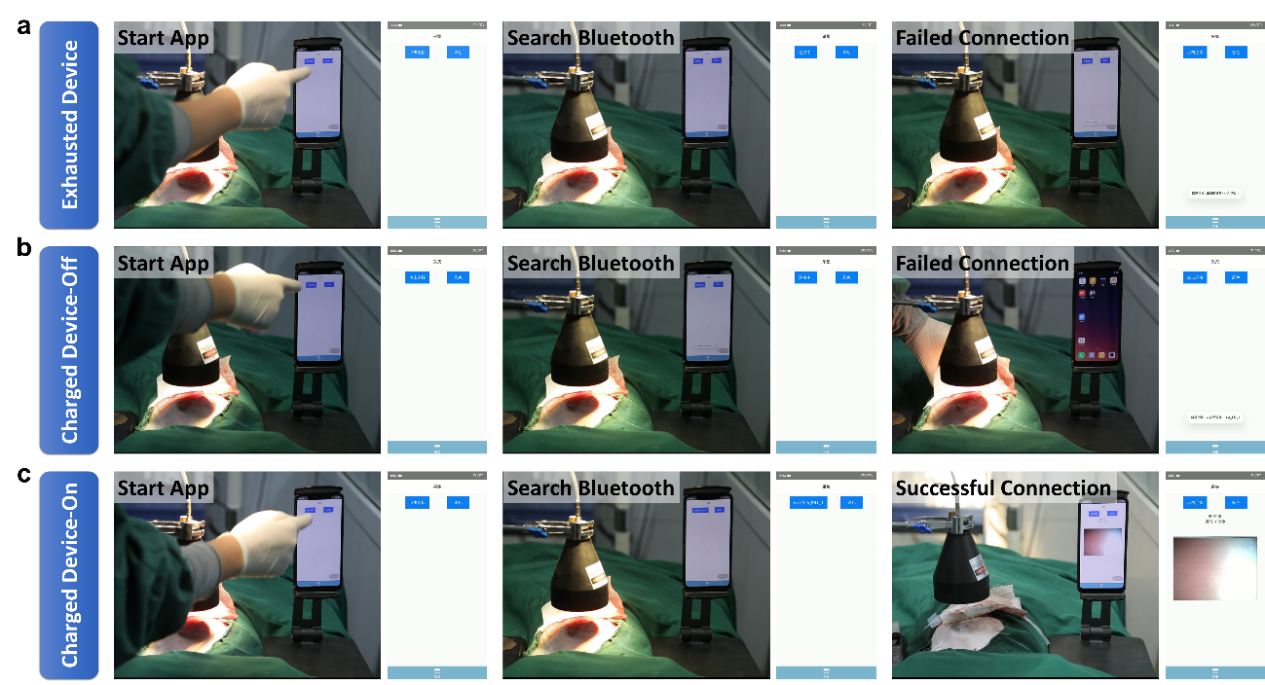
**

**Supplementary Fig. 43 | Indirect power supply for an implanted wireless camera by Bio-PS. a,** The battery of the wireless camera had run out before implanting, and the camera was unable to link the app. **b,** The wireless camera and app had been restarted, which reconfirmed that it was unable to link. **c,** After charging for 2 hours by Bio-PS, the wireless camera and app restarted again, and the phone could receive the video signals via Bluetooth.

**
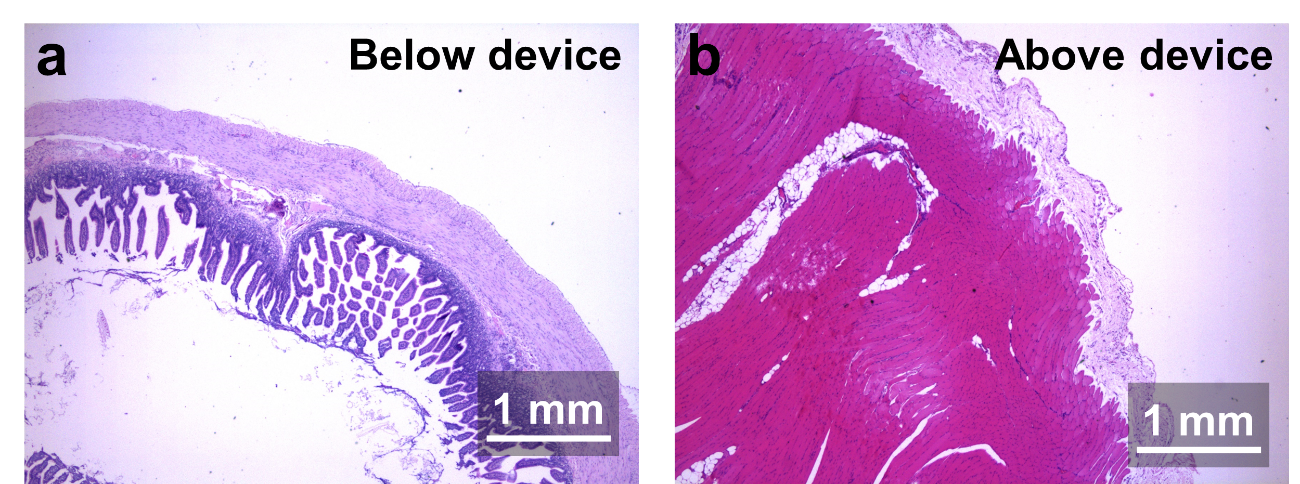
**

**Supplementary Fig. 44 |** **Standard H&E staining of abdomen tissues after 31 days since the implanted surgery. a,** The tissue below the implanted Bio-PS. **b,** The tissue above the implanted Bio-PS. Scale bar: 1.0 mm. (n=3)

**4. References for Supporting Information**

1. Rich, B.R. An investigation of heat transfer from an inclined flat plate in free convection. *Trans. ASME.* **75,** 489-499 (1953).
2. Gryzagoridis, J. Leading edge effects on the nusselt number for a vertical plate in free convection. *Int. J. Heat Mass Trans.* **16,** 517-520 (1973).
3. Black, W.Z., Jack, K.N. The thermal structure of free convection turbulence from inclined isothermal surfaces and its influence on heat transfer. *Int. J. Heat Mass Trans.* **18(1),** 43-50 (1975).
4. Vliet, G. C., Liu, C. K. An experimental study of turbulent natural convection boundary layers.  *J. Heat Trans.* **91(4),** 517-531 (1969).
5. Fujii, T. Experimental studies of free conviction heat transfer. *Bull. JSME,* **2(8),** 555-558 (1959).
6. Yang, S.M., Tao, W.Q. Heat transfer theory. Higher Education Press. 4th ed (2006).
7. Holman, J. P. Heat transfer. Boston McGraw Hill Higher Education. 10th ed (2010).
8. He, Y., Xu, X.-Q., Lv, S., Liao, H. & Wang, Y. Dark ionic liquid for flexible optoelectronics. *Langmuir* **35**, 1192-1198 (2019).
9. Lyu, S., He, Y., Yao, Y., Zhang, M. & Wang, Y. Photothermal clothing for thermally preserving pipeline transportation of crude oil. *Adv. Funct. Mater.* **29**, 1900703 (2019).
10. Lu, L. et al. Biodegradable monocrystalline silicon photovoltaic microcells as power supplies for transient biomedical implants. *Adv. Energy Mater.* **8**, 1703035 (2018).
11. Ho, J.S. et al. Wireless power transfer to deep-tissue microimplants. *P. Natl. Acad. Sci. USA* **111,** 7974-7979 (2014).
12. Song, K. et al. Subdermal flexible solar cell arrays for powering medical electronic implants. *Adv. Healthc. Mater.* **5**, 1572-1580 (2016).
13. Basaeri, H., Yu, Y., Young, D. & Roundy, S. A MEMS-scale ultrasonic power receiver for biomedical implants. *IEEE Sens. Lett.* **3**, 1-4 (2019).
14. Wu, T., Redouté, J. & Yuce, M.R. A wireless implantable sensor design with subcutaneous energy harvesting for long-term IoT healthcare applications. *IEEE Access* **6**, 35801-35808 (2018).
15. Haeberlin, A. et al. Successful pacing using a batteryless sunlight-powered pacemaker. *Europace* **16**, 1534-1539 (2014).
16. Kim, J. et al. Active photonic wireless power transfer into live tissues. *P. Natl. Acad. Sci. USA* 117, 16856-16863 (2020).
17. Chen, Z., Zhang, L., Sun, Y., Hu, J. & Wang, D. 980-nm Laser-Driven photovoltaic cells based on rare-earth up-converting phosphors for biomedical applications. *Adv. Funct. Mater.* **19**, 3815-3820 (2009).
18. Moon, E., Blaauw, D. & Phillips, J.D. Subcutaneous photovoltaic infrared energy harvesting for bio-implantable devices. *IEEE T. Electron Dev.* **64**, 2432-2437 (2017).
19. Chen, Z., Law, M., Mak, P. & Martins, R.P. A single-chip solar energy harvesting IC using integrated photodiodes for biomedical implant applications. *IEEE T. Biomed. Circ. S.* **11**, 44-53 (2017).
20. Ayazian, S., Akhavan, V.A., Soenen, E. & Hassibi, A. A photovoltaic-driven and energy-autonomous CMOS implantable sensor. *IEEE T. Biomed. Circ. S.* **6**, 336-343 (2012).
21. Hung, Y. et al. High-voltage backside-illuminated CMOS photovoltaic module for powering implantable temperature sensors. *IEEE J. Photovolt.* **8**, 342-347 (2018).
22. Li, N. et al. Direct powering a real cardiac pacemaker by natural energy of a heartbeat. *ACS Nano* **13**, 2822-2830 (2019).
23. El Ichi, S. et al. Bioelectrodes modified with chitosan for long-term energy supply from the body. *Energ. Environ. Sci.* **8**, 1017-1026 (2015).
24. Schroeder, T.B.H. et al. An electric-eel-inspired soft power source from stacked hydrogels. *Nature* **552**, 214-218 (2017).
25. Mercier, P.P., Lysaght, A.C., Bandyopadhyay, S., Chandrakasan, A.P. & Stankovic, K.M. Energy extraction from the biologic battery in the inner ear. *Nat. Biotechnol.* **30**, 1240-1243 (2012).
26. Kim, D.H. et al. In vivo self-powered wireless transmission using biocompatible flexible energy harvesters. *Adv. Funct. Mater.* **27**, 1700341 (2017).
