# Supplementary material for: Subcutaneous power supply by NIR-II light
Source: Nat Commun. 2022 Nov 3;13:6596. doi: 10.1038/s41467-022-34047-5 (PMC9633840; doi:10.1038/s41467-022-34047-5)
Supplement: Supplementary file 2 — Description of Additional Supplementary Files [file 41467_2022_34047_MOESM2_ESM.docx]

**Description of Additional Supplementary Files**

**Supplementary Movie 1:** FEMs of the none-heat transfer model. This video describes the influence of boundary condition (including *H*_1_, *H*_2_ and *P*_PT_) on simulated temperature difference between TE generator (*t*_1_-*t*_2_).

**Supplementary Movie 2:** FEMs of the upper layer. This video describes the effect of the thickness of air layer (*d*) on the improvement of energy conversion and the safety of the device.

**Supplementary Movie 3:** FEMs of the bottom layer. This video specifies the necessity of using modify phase change materials and fin.

**Supplementary Movie 4:** This video demonstrates the ability of Bio-PS to directly power 648 white LEDs.

**Supplementary Movie 5:** This video demonstrates that the Bio-PS could serve as an indirect power supply to run a camera.

**Supplementary Movie 6:** This video demonstrates that the battery could be recharged by Bio-PS under NIR-II light irradiation, and then drive an electric fan.

**Supplementary Movie 7:** This video describes that the high-frequency cardiac pacemaker implanted in rabbit’s abdominal cavity could be directly powered by Bio-PS under NIR-II light irradiation in vivo. The change of rabbit’s electrocardiogram under light irradiation was an indicator of the successful power supply by Bio-PS.

**Supplementary Movie 8:** This video describes that the battery of a millimeter-sized camera in rabbit’s abdominal cavity could be indirectly recharged by Bio-PS under NIR-II light irradiation in vivo. As consequence, the images captured by the camera in vivo were externally received by a mobile phone via Bluetooth.

**Supplementary Movie 9:** This video describes that the rabbit survived well during the one-month observation without appearance of nursing complications after the implanted surgery.
